# A Mathematical Model with Quarantine States for the Dynamics of Ebola Virus Disease in Human Populations

**DOI:** 10.1155/2016/9352725

**Published:** 2016-08-07

**Authors:** Gideon A. Ngwa, Miranda I. Teboh-Ewungkem

**Affiliations:** ^1^Department of Mathematics, University of Buea, P.O. Box 63, Buea, Cameroon; ^2^Department of Mathematics, Lehigh University, Bethlehem, PA 18015, USA

## Abstract

A deterministic ordinary differential equation model for the dynamics and spread of Ebola Virus Disease is derived and studied. The model contains quarantine and nonquarantine states and can be used to evaluate transmission both in treatment centres and in the community. Possible sources of exposure to infection, including cadavers of Ebola Virus victims, are included in the model derivation and analysis. Our model's results show that there exists a threshold parameter, *R*_0_, with the property that when its value is above unity, an endemic equilibrium exists whose value and size are determined by the size of this threshold parameter, and when its value is less than unity, the infection does not spread into the community. The equilibrium state, when it exists, is locally and asymptotically stable with oscillatory returns to the equilibrium point. The basic reproduction number, *R*_0_, is shown to be strongly dependent on the initial response of the emergency services to suspected cases of Ebola infection. When intervention measures such as quarantining are instituted fully at the beginning, the value of the reproduction number reduces and any further infections can only occur at the treatment centres. Effective control measures, to reduce *R*_0_ to values below unity, are discussed.

## 1. Introduction and Background

The world has been riveted by the 2014 outbreak of the Ebola Virus Disease (EVD) that affected parts of West Africa with Guinea, Liberia, and Sierra Leone being the most hard hit areas. Isolated cases of the disease did spread by land to Senegal and Mali (localized transmission) and by air to Nigeria. Some Ebola infected humans were transported to the US (except the one case that traveled to Texas and later on died) and other European countries for treatment. An isolated case occurred in Spain, another in Italy (a returning volunteer health care worker), and a few cases in the US and the UK [1–3]. Though dubbed the West African Ebola outbreak, the movement of patients and humans between countries, if not handled properly, could have led to a global Ebola pandemic. There was also a separate Ebola outbreak affecting a remote region in the Democratic Republic of Congo (formerly Zaire), and it was only by November 21, 2014 that the outbreak was reported to have ended [2].

The Ebola Virus Disease (EVD), formally known as Ebola haemorrhagic fever and caused by the Ebola Virus, is very lethal with case fatalities ranging from 25% to 90%, with a mean of about 50% [2]. The 2014 EVD outbreak, though not the first but one of many other EVD outbreaks that have occurred in Africa since the first recorded outbreak of 1976, is the worst in terms of the numbers of Ebola cases and related deaths and the most complex [2]. About 9 months after the identification of a mysterious killer disease killing villagers in a small Guinean village as Ebola, the 2014 West African Ebola outbreak, as of December 24, 2014, had up to 19497 Ebola cases resulting in 7588 fatalities [1, 3, 4], a case fatality rate of about 38.9%. By December 2015, the number of Ebola Virus cases (including suspected, probable, and confirmed) stood at 28640 resulting in 11315 fatalities, a case fatality rate of 39.5% [3, 5].

Ebola Virus, the agent that causes EVD, is hypothesised to be introduced into the human population through contact with the blood, secretions, fluids from organs, and other body parts of dead or living animals infected with the virus (e.g., fruit bats, primates, and porcupines) [2, 6]. Human-to-human transmission can then occur through direct contact (via broken skin or mucous membranes such as eyes, nose, or mouth) with Ebola Virus infected blood, secretions, and fluids secreted through organs or other body parts, in, for example, saliva, vomit, urine, faeces, semen, sweat, and breast milk. Transmission can also be as a result of indirect contact with surfaces and materials, in, for example, bedding, clothing, and floor areas, or objects such as syringes, contaminated with the aforementioned fluids [2, 6].

When a healthy human (considered here to be susceptible) who has no Ebola Virus in them is exposed to the virus (directly or indirectly), the human may become infected, if transmission is successful. The risk of being infected with the Ebola Virus is (i) very low or not recognizable where there is casual contact with a feverish, ambulant, self-caring patient, for example, sharing the same public place, (ii) low where there is close face-to-face contact with a feverish and ambulant patient, for example, physical examination, (iii) high where there is close face-to-face contact without appropriate personal protective equipment (including eye protection) with a patient who is coughing or vomiting, has nosebleeds, or has diarrhea, and (iv) very high where there is percutaneous, needle stick, or mucosal exposure to virus-contaminated blood, body fluids, tissues, or laboratory specimens in severely ill or known positive patients. The symptoms of EVD may appear during the incubation period of the disease, estimated to be anywhere from 2 to 21 days [2, 7–9], with an estimated 8- to 10-day average incubation period, although studies estimate it at 9–11 days for the 2014 EVD outbreak in West Africa [10]. Studies have shown that, during the asymptomatic part of the Ebola Virus Disease, a human infected with the virus is not infectious and cannot transmit the virus. However, with the onset of symptoms, the human can transmit the virus and is hence infectious [2, 7]. The onset of symptoms commences the course of illness of the disease which can lead to death 6–16 days later [8, 9] or improvement of health with evidence of recovery 6–11 days later [8].

In the first few days of EVD illness (estimated at days 1–3 [11]), a symptomatic patient may exhibit symptoms common to those like the malaria disease or the flu (high fever, headache, muscle and joint pains, sore throat, and general weakness). Without effective disease management, between days 4 and 5 to 7, the patient progresses to gastrointestinal symptoms such as nausea, watery diarrhea, vomiting, and abdominal pain [10, 11]. Some or many of the other symptoms, such as low blood pressure, headaches, chest pain, shortness of breath, anemia, exhibition of a rash, abdominal pain, confusion, bleeding, and conjunctivitis, may develop [10, 11] in some patients. In the later phase of the course of the illness, days 7–10, patients may present with confusion and may exhibit signs of internal and/or visible bleeding, progressing towards coma, shock, and death [10, 11].

Recovery from EVD can be achieved, as evidenced by the less than 50% fatality rate for the 2014 EVD outbreak in West Africa. With no known cure, recovery is possible through effective disease management, the treatment of Ebola-related symptoms, and also the effective protection by the patient's immune response [7]. Some of the disease management strategies include hydrating patients by administering intravenous fluids and balancing electrolytes and maintaining the patient's blood pressure and oxygen levels. Other schemes used include blood transfusion (using an Ebola survivor's blood) and the use of experimental drugs on such patients (e.g., ZMAPP whose safety and efficacy have not yet been tested on humans). There are some other promising drugs/vaccines under trials [2]. Studies show that once a patient recovers from EVD they remain protected against the disease and are immune to it at least for a projected period because they develop antibodies that last for at least 10 years [7]. Once recovered, lifetime immunity is unknown or whether a recovered individual can be infected with another Ebola strain is unknown. However, after recovery, a person can potentially remain infectious as long as their blood and body fluids, including semen and breast milk, contain the virus. In particular, men can potentially transmit the virus through their seminal fluid, within the first 7 to 12 weeks after recovery from EVD [2].  Table 1 shows the estimated time frames and projected progression of the infection in an average EVD patient.

Given that there is no approved drug or vaccine out yet, local control of the Ebola Virus transmission requires a combined and coordinated control effort at the individual level, the community level, and the institutional/health/government level. Institutions and governments need to educate the public and raise awareness about risk factors, proper hand washing, proper handling of Ebola patients, quick reporting of suspected Ebola cases, safe burial practices, use of public transportation, and so forth. These education efforts need to be communicated with community/chief leaders who are trusted by members of the communities they serve. From a global perspective, a good surveillance and contact tracing program followed by isolation and monitoring of probable and suspected cases, with immediate commencement of disease management for patients exhibiting symptoms of EVD, is important if we must, in the future, elude a global epidemic and control of EVD transmission locally and globally [2]. It was by effective surveillance, contact tracing, and isolation and monitoring of probable and suspected cases followed by immediate supportive care for individuals and families exhibiting symptoms that the EVD was brought under control in Nigeria [17], Senegal, USA, and Spain [1].

Efficient control and management of any future EVD outbreaks can be achieved if new, more economical, and realizable methods are used to target and manage the dynamics of spread as well as the population sizes of those communities that may be exposed to any future Ebola Virus Disease outbreak. More realistic mathematical models can play a role in this regard, since analyses of such models can produce clear insight to vulnerable spots on the Ebola transmission chain where control efforts can be concentrated. Good models could also help in the identification of disease parameters that can possibly influence the size of the reproduction number of EVD. Existing mathematical models for Ebola [14, 16, 18–21] have been very instrumental in providing mathematical insight into the dynamics of Ebola Virus transmission. Many of these models have also been helpful in that they have provided methods to derive estimates for the reproduction number for Ebola based on data from the previous outbreaks. However, few of the models have taken into account the fact that institution of quarantine states or treatment centres will affect the course of the epidemic in the population [16]. It is our understanding that the way the disease will spread will be determined by the initial and continual response of the health services in the event of the discovery of an Ebola disease case. The objective of this paper is to derive a comprehensive mathematical model for the dynamics of Ebola transmission taking into consideration what is currently known of the disease. The primary objective is to derive a formula for the reproduction number for Ebola Virus Disease transmission in view of providing a more complete and measurable index for the spread of Ebola and to investigate the level of impact of surveillance, contact tracing, isolation, and monitoring of suspected cases, in curbing disease transmission. The model is formulated in a way that it is extendable, with appropriate modifications, to other disease outbreaks with similar characteristics to Ebola, requiring such contact tracing strategies. Our model differs from other mathematical models that have been used to study the Ebola disease [14, 15, 18, 20–22] in that it captures the quarantined Ebola Virus Disease patients and provides possibilities for those who escaped quarantine at the onset of the disease to enter quarantine at later stages. To the best of our knowledge, this is the first integrated ordinary differential equation model for this kind of communicable disease of humans. Our final result would be a formula for the basic reproduction number of Ebola that depends on the disease parameters.

The rest of the paper is divided up as follows. In Section 2, we outline the derivation of the model showing the state variables and parameters used and how they relate together in a conceptual framework. In Section 3, we present a mathematical analysis of the derived model to ascertain that the results are physically realizable. We then reparameterise the model and investigate the existence and linear stability of steady state solution, calculate the basic reproduction number, and present some special cases. In Section 4, we present a discussion on the parameters of the model. In Section 5, we carry out some numerical simulations based on the selected feasible parameters for the system and then round up the paper with a discussion and conclusion in Section 6.

## 2. The Mathematical Model

### 2.1. Description of Model Variables

We divide the human population into 11 states representing disease status and quarantine state. At any time *t* there are the following.

*(1) Susceptible Individuals.* Denoted by *S*, this class also includes false probable cases, that is, all those individuals who would have displayed early Ebola-like symptoms but who eventually return a negative test for Ebola Virus infection.

*(2) Suspected Ebola Cases*. The class of suspected EVD patients comprises those who have come in contact with, or been in the vicinity of, anybody who is known to have been sick or died of Ebola. Individuals in this class may or may not show symptoms. Two types of suspected cases are included: the quarantined suspected cases, denoted by *S*_*Q*_, and the nonquarantined suspected case, denoted by *S*_*N*_. Thus a suspected case is either quarantined or not. 

*(3) Probable Cases*. The class of probable cases comprises all those persons who at some point were considered suspected cases and who now present with fever and at least three other early Ebola-like symptoms. Two types of probable cases are included: the quarantined probable cases, denoted by *P*_*Q*_, and the nonquarantined probable cases, *P*_*N*_. Thus a probable case is either quarantined or not. Since the early Ebola-like symptoms of high fever, headache, muscle and joint pains, sore throat, and general weakness can also be a result of other infectious diseases such as malaria or flu, we cannot be certain at this stage whether or not the persons concerned have Ebola infection. However, since the class of probable persons is derived from suspected cases, and to remove the uncertainties, we will assume that probable cases may eventually turn out to be EVD patients and if that were to be the case, since they are already exhibiting some symptoms, they can be assumed to be mildly infectious.

*(4) Confirmed Early Symptomatic Cases*. The class of confirmed early asymptomatic cases comprises all those persons who at some point were considered probable cases and a confirmatory laboratory test has been conducted to confirm that there is indeed an infection with Ebola Virus. This class is called confirmed early symptomatic because all that they have as symptoms are the early Ebola-like symptoms of high fever, headache, muscle and joint pains, sore throat, and general weakness. Two types of confirmed early symptomatic cases are included: the quarantined confirmed early symptomatic cases *C*_*Q*_ and the nonquarantined confirmed early symptomatic cases *C*_*N*_. Thus a confirmed early symptomatic case is either quarantined or not. The class of confirmed early symptomatic individuals may not be very infectious.

*(5) Confirmed Late Symptomatic Cases*. The class of confirmed late symptomatic cases comprises all those persons who at some point were considered confirmed early symptomatic cases and in addition the persons who now present with most or all of the later Ebola-like symptoms of vomiting, diarrhea, stomach pain, skin rash, red eyes, hiccups, internal bleeding, and external bleeding. Two types of confirmed late symptomatic cases are included: the quarantined confirmed late symptomatic cases *I*_*Q*_ and the nonquarantined confirmed late symptomatic cases *I*_*N*_. Thus a confirmed late symptomatic case is either quarantined or not. The class of confirmed late symptomatic individuals may be very infectious and any bodily secretions from this class of persons can be infectious to other humans. 

*(6) Removed Individuals*. Three types of removals are considered, but only two are related to EVD. The removals related to the EVD are confirmed individuals removed from the system through disease induced death, denoted by *R*_*D*_, or confirmed cases that recover from the infection denoted by *R*_*R*_. Now, it is known that unburied bodies or not yet cremated cadavers of EVD victims can infect other susceptible humans upon contact [7]. Therefore, the cycle of infection really stops only when a cadaver is properly buried or cremated. Thus members from class, *R*_*D*_, representing dead bodies or cadavers of EVD victims are considered removed from the infection chain, and consequently from the system, only when they have been properly disposed of. The class, *R*_*R*_, of individuals who beat the odds and recover from their infection are considered removed because recovery is accompanied with the acquisition of immunity so that this class of individuals are then protected against further infection [7] and they no longer join the class of susceptible individuals. The third type of removal is obtained by considering individuals who die naturally or due to other causes other than EVD. These individuals are counted as *R*_*N*_.

The state variables are summarized in Notations.

### 2.2. The Mathematical Model

A compartmental framework is used to model the possible spread of EVD within a population. The model accounts for contact tracing and quarantining, in which individuals who have come in contact or have been associated with Ebola infected or Ebola-deceased humans are sought and quarantined. They are monitored for twenty-one days during which they may exhibit signs and symptoms of the Ebola Virus or are cleared and declared free. We assume that most of the quarantining occurs at designated makeshift, temporal, or permanent health facilities. However, it has been documented that others do not get quarantined, because of fear of dying without a loved one near them or fear that if quarantined they may instead get infected at the centre, as well as traditional practices and belief systems [14, 16, 22]. Thus, there may be many within communities who remain nonquarantined, and we consider these groups in our model. In all the living classes discussed, we will assume that natural death, or death due to other causes, occurs at constant rate *μ* where 1/*μ* is approximately the life span of the human.

#### 2.2.1. The Susceptible Individuals

The number of susceptible individuals in the population decreases when this population is exposed by having come in contact with or being associated with any of the possibly infectious cases, namely, infected probable case, confirmed case, or the cadaver of a confirmed case. The density increases when some false suspected individuals (a proportion of 1 − *θ*_2_ of nonquarantined and 1 − *θ*_6_ of quarantined) and probable cases (a proportion of 1 − *θ*_3_ of nonquarantined individuals and 1 − *θ*_7_ of quarantined individuals) are eliminated from the suspected and probable case list. We also assume a constant recruitment rate Π as well as natural death, or death due to other causes. Therefore the equation governing the rate of change with time within the class of susceptible individuals may be written as

(1) dSdt=Π−λS+1−θ3βNPN+1−θ2αNSN+1−θ6αQSQ+1−θ7βQPQ−μS,

where *λ* is the force of infection and the rest of the parameters are positive and are defined in Notations. We identify two types of total populations at any time *t*: (i) the total living population, *H*_*L*_, and (ii) the total living population including the cadavers of Ebola Virus victims that can take part in the spread of EVD, *H*. Thus at each time *t* we have

(2) HLt=S+SN+SQ+PN+PQ+CN+CQ+IN+IQ+RRt,

(3) Ht=S+SN+SQ+PN+PQ+CN+CQ+IN+IQ+RR+RDt.

Since the cadavers of EVD victims that have not been properly disposed of are very infectious, the force of infection must then also take this fact into consideration and be weighted with *H* instead of *H*_*L*_. The force of infection takes the following form:

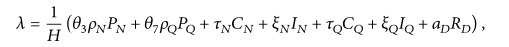

(4)
where *H* > 0 is defined above and the parameters *ρ*_*N*_, *ρ*_*Q*_, *τ*_*N*_, *τ*_*Q*_, *ξ*_*N*_, *ξ*_*Q*_, and *a*_*D*_ are positive constants as defined in Notations. There are no contributions to the force of infection from the *R*_*R*_ class because it is assumed that once a person recovers from EVD infection, the recovered individual acquires immunity to subsequent infection with the same strain of the virus. Although studies have suggested that recovered men can potentially transmit the Ebola Virus through seminal fluids within the first 7–12 weeks of recovery [2], and mothers through breast milk, we assume, here, that, with education, survivors who recover would have enough information to practice safe sexual and/or feeding habits to protect their loved ones until completely clear. Thus recovered individuals are considered not to contribute to the force of infection.

#### 2.2.2. The Suspected Individuals

A fraction 1 − *θ*_1_ of the exposed susceptible individuals get quarantined while the remaining fraction are not. Also, a fraction *θ*_2_ (resp., *θ*_6_) of the nonquarantined (resp., quarantined) suspected individuals become probable cases at rate *α* while the remainder 1 − *θ*_2_ (resp., 1 − *θ*_6_) do not develop into probable cases and return to the susceptible pool. For the quarantined individuals, we assume that they are being monitored, while the suspected nonquarantined individuals are not. However, as they progress to probable cases (at rates *α*_*N*_ and *α*_*Q*_), a fraction *θ*_2*b*_ of these humans will seek the health care services as symptoms commence and become quarantined while the remainder *θ*_2*a*_ remain nonquarantined. Thus the equation governing the rate of change within the two classes of suspected persons then takes the following form:

(5) dSNdt=θ1λS−1−θ2αNSN−θ2aαNSN−θ2bαNSN−μSN,dSQdt=1−θ1λS−1−θ6αQSQ−θ6αQSQ−μSQ.

In the context of this model we make the assumption that once quarantined, the individuals stay quarantined until clearance and are released, or they die of the infection. Notice that *θ*_2_ = *θ*_2*a*_ + *θ*_2*b*_, so that 1 − *θ*_2_ + *θ*_2*a*_ + *θ*_2*b*_ = 1.

#### 2.2.3. The Probable Cases

The fractions *θ*_2_ and *θ*_6_ of suspected cases that become probable cases increase the number of individuals in the probable case class. The population of probable cases is reduced (at rates *β*_*N*_ and *β*_*Q*_) when some of these are confirmed to have the Ebola Virus through laboratory tests at rates *α*_*N*_ and *α*_*Q*_. For some, proportions 1 − *θ*_3_ and 1 − *θ*_7_, the laboratory tests are negative and the probable individuals revert to the susceptible class. From the proportion *θ*_3_ of nonquarantined probable cases whose tests are positive for the Ebola Virus (i.e., confirmed for EVD), a fraction, *θ*_3*b*_, become quarantined while the remainder, *θ*_3*a*_, remain nonquarantined. So *θ*_3_ = *θ*_3*a*_ + *θ*_3*b*_. Thus the equation governing the rate of change within the classes of probable cases takes the following form:

(6) dPNdt=θ2aαNSN−1−θ3βNPN−θ3aβNPN−θ3bβNPN−μPN,dPQdt=θ2bαNSN+θ6αQSQ−1−θ7βQPQ−θ7βQPQ−μPQ.

#### 2.2.4. The Confirmed Early Symptomatic Cases

The fractions *θ*_3_ and *θ*_7_ of the probable cases become confirmed early symptomatic cases thus increasing the number of confirmed cases with early symptoms. The population of early symptomatic individuals is reduced when some recover at rates *r*_*EN*_ for the nonquarantined cases and *r*_*EQ*_ for the quarantined cases. Others may see their condition worsening and progress and become late symptomatic individuals, in which case they enter the full blown late symptomatic stages of the disease. We assume that this progression occurs at rates *γ*_*N*_ or *γ*_*Q*_, respectively, which are the reciprocal of the mean time it takes for the immune system to either be completely overwhelmed by the virus or be kept in check via supportive mechanism. A fraction 1 − *θ*_4_ of the confirmed nonquarantined early symptomatic cases will be quarantined as they become late symptomatic cases, while the remaining fraction *θ*_4_ escape quarantine due to lack of hospital space or fear and belief customs [16, 22] but become confirmed late symptomatic cases in the community. Thus the equation governing the rate of change within the two classes of confirmed early symptomatic cases takes the following form:

(7) dCNdt=θ3aβNPN−rENCN−θ4γNCN−1−θ4γNCN−μCN,dCQdt=θ3bβNPN+θ7βQPQ−rEQCQ−γQCQ−μCQ.

#### 2.2.5. The Confirmed Late Symptomatic Cases

The fractions *θ*_4_ and *θ*_8_ of confirmed early symptomatic cases who progress to the late symptomatic stage increase the number of confirmed late symptomatic cases. The population of late symptomatic individuals is reduced when some of these individuals are removed. Removal could be as a result of recovery at rates proportional to *r*_*LN*_ and *r*_*LQ*_ or as a result of death because the EVD patient's conditions worsen and the Ebola Virus kills them. The death rates are assumed proportional to *δ*_*N*_ and *δ*_*Q*_. Additionally, as a control effort or a desperate means towards survival, some of the nonquarantined late symptomatic cases are removed and quarantined at rate *σ*_*N*_. In our model, we assume that Ebola-related death only occurs at the late symptomatic stage. Additionally, we assume that the confirmed late symptomatic individuals who are eventually put into quarantine at this late period (removing them from the community) may not have long to live but may have a slightly higher chance at recovery than when in the community and nonquarantined. Since recovery confers immunity against the particular strain of the Ebola Virus, individuals who recover become refractory to further infection and hence are removed from the population of susceptible individuals. Thus the equation governing the rate of change within the two classes of confirmed late symptomatic cases takes the following form:

(8) dINdt=θ4γNCN−δNIN−σNIN−rLNIN−μIN,dIQdt=1−θ4γNCN+σNIN+γQCQ−δQIQ−rLQIQ−μIQ.

#### 2.2.6. The Cadavers and the Recovered Persons

The dead bodies of EVD victims are still very infectious and can still infect susceptible individuals upon effective contact [2]. Disease induced deaths from the class of confirmed late symptomatic individuals occur at rates *δ*_*N*_ and *δ*_*Q*_ and the cadavers are disposed of via burial or cremation at rate *b*. The recovered class contains all individuals who recover from EVD. Since recovery is assumed to confer immunity against the 2014 strain (the Zaire Virus) [7] of the Ebola Virus, once an individual recovers, they become removed from the population of susceptible individuals. Thus the equation governing the rate of change within the two classes of recovered persons and cadavers takes the following form:

(9) dRRdt=rENCN+rLNIN+rEQCQ+rLQIQ−μIN,dRDdt=δNIN+δQIQ−bRD.

The population of humans who die either naturally or due to other causes is represented by the variable *R*_*N*_ and keeps track of all natural deaths, occurring at rate *μ*, from all the living population classes. This is a collection class. Another collection class is the class of disposed Ebola-related cadavers, disposed at rate *b*. These collection classes satisfy the equations

(10) dRNdt=μHL,dDDdt=bRD.

Putting all the equations together we have

(11) dSdt=Π−λS+θ~3βNPN+θ~2αNSN+θ~6αQSQ+θ~7βQPQ−μS,

(12) dSNdt=θ1λS−αN+μSN,

(13) dSQdt=θ~1λS−αQ+μSQ,

(14) dPNdt=θ2aαNSN−βN+μPN,

(15) dPQdt=θ2bαNSN+θ6αQSQ−βQ+μPQ,

(16) dCNdt=θ3aβNPN−rEN+γN+μCN,

(17) dCQdt=θ3bβNPN+θ7βQPQ−rEQ+γQ+μCQ,

(18) dINdt=θ4γNCN−rLN+δN+μIN,

(19) dIQdt=θ~4γNCN+σNIN+γQCQ−rLQ+δQ+μIQ,

(20) dRRdt=rENCN+rLNIN+rEQCQ+rLQIQ−μRR,

(21) dRDdt=δNIN+δQIQ−bRD,

(22) dRNdt=μHL

(23) dDDdt=bRD,

where ${\tilde{\theta }}_{*}=1-\theta_{*}$ and all other parameters and state variables are as in Notations.

Suitable initial conditions are needed to completely specify the problem under consideration. We can, for example, assume that we have a completely susceptible population, and a number of infectious persons are introduced into the population at some point. We can, for example, have that

(24) S0S0,IN0I0,SN0SQ0=PN0=0,PQ0CN0=CQ0=IQ0=RD0=RR0=RN0=DD0=0.

Class *D*_*D*_ is used to keep count of all the dead that are properly disposed of, class *R*_*D*_ is used to keep count of all the deaths due to EVD, and class *R*_*N*_ is used to keep count of the deaths due to causes other than EVD infection. The rate of change equation for the two groups of total populations is obtained by using (2) and (3) and adding up the relevant equations from (11) to (23) to obtain

(25) dHLdt=Π−μHL−δNIN−δQIQ,

(26) dHdt=Π−μH−b−μRD,

where *H*_*L*_ is the total living population and *H* is the augmented total population adjusted to account for nondisposed cadavers that are known to be very infectious. On the other hand, if we keep count of all classes by adding up (11)–(23), the total human population (living and dead) will be constant if Π = 0. In what follows, we will use the classes *R*_*D*_, *R*_*N*_, and *D*_*D*_, comprising classes of already dead persons, only as place holders, and study the problem containing the living humans and their possible interactions with cadavers of EVD victims as often is the case in some cultures in Africa, and so we cannot have a constant total population. Note that (26) can also be written as *dH*/*dt* = Π − *μH*_*L*_ − *bR*_*D*_.

#### 2.2.7. Infectivity of Persons Infected with EVD

Ebola is a highly infectious disease and person to person transmission is possible whenever a susceptible person comes in contact with bodily fluids from an individual infected with the Ebola Virus. We therefore define effective contact here generally to mean contact with these fluids. The level of infectivity of an infected person usually increases with duration of the infection and severity of symptoms and the cadavers of EVD victims are the most infectious [23]. Thus we will assume in this paper that probable persons who indeed are infected with the Ebola Virus are the least infectious while confirmed late symptomatic cases are very infectious and the level of infectivity will culminate with that of the cadaver of an EVD victim. While under quarantine, it is assumed that contact between the persons in quarantine and the susceptible individuals is minimal. Thus though the potential infectivity of the corresponding class of persons in quarantine increases with disease progression, their effective transmission to members of the public is small compared to that from the nonquarantined class. It is therefore reasonable to assume that any transmission from persons under quarantine will affect mostly health care providers and use that branch of the dynamics to study the effect of the transmission of the infections to health care providers who are here considered part of the total population. In what follows we do not explicitly single out the infectivity of those in quarantine but study general dynamics as derived by the current modelling exercise.

## 3. Mathematical Analysis

### 3.1. Well-Posedness, Positivity, and Boundedness of Solution

In this subsection we discuss important properties of the model such as well-posedness, positivity, and boundedness of the solutions. We start by defining what we mean by a realistic solution.

**Definition 1 (realistic solution).**

A solution of system (27) or equivalently system comprising (11)–(21) is called realistic if it is nonnegative and bounded.

It is evident that a solution satisfying Definition 1 is physically realizable in the sense that its values can be measured through data collection. For notational simplicity, we use vector notation as follows: let **x** = (*S*, *S*_*N*_, *S*_*Q*_, *P*_*N*_, *P*_*Q*_, *C*_*N*_, *C*_*Q*_, *I*_*N*_, *I*_*Q*_, *R*_*R*_, *R*_*D*_)^*T*^ be a column vector in *ℝ* ^11^ containing the 11 state variables, so that, in this notation, *x*_1_ = *S*, *x*_2_ = *S*_*N*_,…, *x*_11_ = *R*_*D*_. Let **f**(**x**) = (*f*_1_(**x**), *f*_2_(**x**),…, *f*_11_(**x**))^*T*^ be the vector valued function defined in *ℝ* ^11^ so that in this notation *f*_1_(**x**) is the right-hand side of the differential equation for first variable *S*, *f*_2_(**x**) is the right-hand side of the equation for the second variable *x*_2_ = *S*_*N*_,…, and *f*_11_(**x**) is the right side of the differential equation for the 11th variable *x*_11_ = *R*_*D*_, and so is precisely system (11)–(21) in that order with prototype initial conditions (24). We then write the system in the form

(27) $$\begin{array}{c} \frac{dx}{dt}=f\left(\;x\;\right),\;x\left(0\right)=x_{0}, \end{array}$$

where **x** : [0, *∞*) → *ℝ* ^11^ is a column vector of state variables and **f** : *ℝ* ^11^ → *ℝ* ^11^ is the vector containing the right-hand sides of each of the state variables as derived from corresponding equations in (11)–(21). We can then have the following result.

**Lemma 2 .**

The function **f** in (27) is Lipschitz continuous in **x**.

**Proof**

Since all the terms in the right-hand side are linear polynomials or rational functions of nonvanishing polynomial functions, and since the state variables, *S*, *S*_*∗*_, *P*_*∗*_, *C*_*∗*_, *I*_*∗*_, and *R*_*∗*_, are continuously differentiable functions of *t*, the components of the vector valued function **f** of (27) are all continuously differentiable. Further, let **L**(**x**, **y**; *θ*) = {**x** + *θ*(**y** − **x**) : 0 ≤ *θ* ≤ 1}. Then **L**(**x**, **y**; *θ*) is a line segment that joins points **x** to the point **y** as *θ* ranges on the interval [0,1]. We apply the mean value theorem to see that

(28) fy−fx∞=f′z;y−x∞,z∈Lx,y;θ,  a  mean  value  point,

where **f**′(**z**; **y** − **x**) is the directional derivative of the function **f** at the mean value point **z** in the direction of the vector **y** − **x**. But,

(29) f′z;y−x∞∑k=111∇fkz·y−xek∞≤∑k=111∇fkz∞y−x∞,

where **e**_*k*_ is the *k*th coordinate unit vector in *ℝ*_+_^11^. It is now a straightforward computation to verify that since *ℝ*_+_^11^ is a convex set, and taking into consideration the nature of the functions *f*_*i*_, *i* = 1,…, 11, all the partial derivatives are bounded and so there exist *M* > 0 such that

(30) $$\begin{array}{c} {\left\|\;\sum_{k=1}^{11}\nabla f_{k}\left(\;z\;\right)\;\right\|}_{\infty }\leq M\;\forall z\in L\left(x,y;\theta \right)\in R_{+}^{11}, \end{array}$$

and so there exist *M* > 0 such that

(31) $$\begin{array}{c} {\left\|\;f\left(\;y\;\right)-f\left(\;x\;\right)\;\right\|}_{\infty }\leq M{\left\|\;y-x\;\right\|}_{\infty } \end{array}$$

and hence **f** is Lipschitz continuous.

**Theorem 3 (uniqueness of solutions).**

The differential equation (27) has a unique solution.

**Proof**

By Lemma 2, the right-hand side of (27) is Lipschitzian; hence a unique solution exists by existence and uniqueness theorem of Picard. See, for example, [24].

**Theorem 4 (positivity).**

The region *ℝ*_+_^11^ wherein solutions defined by (11)–(21) are defined is positively invariant under the flow defined by that system.

**Proof**

We show that each trajectory of the system starting in *ℝ*_+_^11^ will remain in *ℝ*_+_^11^. Assume for a contradiction that there exists a point *t*_1_ ∈ [0, *∞*) such that *S*(*t*_1_) = 0, *S*′(*t*_1_) < 0 (where the prime denotes differentiation with respect to time) but for 0 < *t* < *t*_1_, *S*(*t*) > 0, and *S*_*N*_(*t*) > 0, *S*_*Q*_(*t*) > 0, *P*_*N*_(*t*) > 0, *P*_*Q*_(*t*) > 0, *C*_*N*_(*t*) > 0, *C*_*Q*_(*t*) > 0, *I*_*N*_(*t*) > 0, *I*_*Q*_(*t*) > 0, *R*_*R*_(*t*) > 0, and *R*_*D*_(*t*) > 0. So, at the point *t* = *t*_1_, *S*(*t*) is decreasing from the value zero in which case it will go negative. If such an *S* will satisfy the given differential equation, then we have

(32) dSdtt=t1Π−λSt1+1−θ3βNPNt1+1−θ2αNSNt1+1−θ6αQSQt1+1−θ7βQPQt1−μSt1=Π+1−θ3βNPNt1+1−θ2αNSNt1+1−θ6αQSQt1+1−θ7βQPQt1>0

and so *S*′(*t*_1_) > 0 contradicting the assumption that *S*′(*t*_1_) < 0. So no such *t*_1_ exist. The same argument can be made for all the state variables. It is now a simple matter to verify, using techniques as explained in [24], that whenever we start system (27), with nonnegative initial data in *ℝ*_+_^11^, the solution will remain nonnegative for all *t* > 0 and that if **x**_0_ = 0, the solution will remain **x** = 0  ∀*t* > 0, and the region *ℝ*_+_^11^ is indeed positively invariant.

The last two theorems have established the fact that, from a mathematical and physical standpoint, the differential equation (27) is well-posed. We next show that the nonnegative unique solutions postulated by Theorem 3 are indeed realistic in the sense of Definition 1.

**Theorem 5 (boundedness).**

The nonnegative solutions characterized by Theorems 3 and 4 are bounded.

**Proof**

It suffices to prove that the total living population size is bounded for all *t* > 0. We show that the solutions lie in the bounded region

(33) $$\begin{array}{c} \Omega_{H_{L}}=\left\{\;H_{L}\left(\;t\;\right):0\leq H_{L}\left(\;t\;\right)\leq \frac{\Pi }{\mu }\;\right\}\subset R_{+}^{11}. \end{array}$$

From the definition of *H*_*L*_ given in (2), if *H*_*L*_ is bounded, the rest of the state variables that add up to *H*_*L*_ will also be bounded. From (25) we have

(34) dHLdt=Π−μHL−δNIN−δQIQ≤Π−μHL⟹HLt≤Πμ+HL0−Πμe−μt.

Thus, from (34), we see that, whatever the size of *H*_*L*_(0), *H*_*L*_(*t*) is bounded above by a quantity that converges to Π/*μ* as *t* → *∞*. In particular, if *μH*_*L*_(0) < Π, then *H*_*L*_(*t*) is bounded above by Π/*μ*, and for all initial conditions

(35) $$\begin{array}{c} H_{L}\left(\;t\;\right)\leq \underset{t\to \infty }{lim}\sup\left(\;\frac{\Pi }{\mu }+\left(\;H_{L}\left(\;0\;\right)-\frac{\Pi }{\mu }\;\right)e^{-\mu t}\;\right). \end{array}$$

Thus *H*_*L*_(*t*) is nonnegative and bounded.

**Remark 6 .**

Starting from the premise that *H*_*L*_(*t*) ≥ 0 for all *t* > 0, Theorem 5 establishes boundedness for the total living population and thus by extension verifies the positive invariance of the positive octant in *ℝ* ^11^ as postulated by Theorem 4, since each of the variables functions *S*, *S*_*∗*_, *P*_*∗*_, *C*_*∗*_, *I*_*∗*_, and *R*_*∗*_, where *∗* ∈ {*N*, *Q*, *R*}, is a subset of *H*_*L*_.

### 3.2. Reparameterisation and Nondimensionalisation

The only physical dimension in our system is that of time. But we have state variables which depend on the density of humans and parameters which depend on the interactions between the different classes of humans. A state variable or parameter that measures the number of individuals of certain type has dimension-like quantity associated with it [25]. To remove the dimension-like character on the parameters and variables, we make the following change of variables:

(36) s=SS0,sn=SNSN0,sq=SQSQ0,pn=PNPN0,pq=PQPQ0,cn=CNCN0,cq=CQCQ0,h=HH0,in=ININ0,iq=IQIQ0,rr=RRRR0,rd=RDRD0,rn=RNRN0,dD=DDDD0,t∗=tT0,hl=HLHL0,

where

(37) S0=Πμ,SN0=SQ0=S0,PN0=θ2aαNSN0βN+μ,PQ0=θ2bαNSN0βQ+μ,CN0=θ3aβNPN0rEN+γN+μ,CQ0=θ3bβNPN0rEQ+γQ+μ,IN0=θ4γNCN0rLN+δN+μ,IQ0=1−θ4γNCN0rLQ+δQ+μ,RR0=rENCN0T0,RD0=δNIN0b,RN0=μT0HL0,DD0=bT0RD0,H0=HL0=Πμ=S0,T0=1μ.

We then define the dimensionless parameter groupings

(38) ρn=θ3ρNPN0μH0,ρq=θ7ρQPQ0μH0,τn=τNCN0μH0,τq=τQCQ0μH0,ξn=ξNIN0μH0,ξq=ξQIQ0μH0,ad=aDRD0μH0,αn=αN+μT0,αq=αQ+μT0,βn=βN+μT0,βq=βQ+μT0,μd=bT0,b1=1−θ3βNPN0μS0,b2=1−θ2αNSN0μS0,b3=1−θ6αQSQ0μS0,b4=1−θ7βQPQ0μS0,b5=δNIN0μH0,b6=δQIQ0μH0,b8=b−μRD0μH0,a1=θ6αQSQ0θ2bαNSN0,a2=θ7βQPQ0θ3bβNPN0,a3=σNIN0rLQ+δQ+μIQ0,a4=γQCQ0rLQ+δQ+μIQ0,a5=rLNIN0rENCN0,a6=rEQCQ0rENCN0,a7=rLQIQ0rENCN0,a8=δQIQ0δNIN0,γn=rEN+γN+μT0,γq=rEQ+γQ+μT0,δq=rLQ+δQ+μT0,δn=rLN+δN+μT0.

The force of infection *λ* then takes the form

(39) λ=ρnpnh+ρqpqh+τncnh+τqcqh+ξninh+ξqiqh+adrdh.

This leads to the equivalent system of equations

(40) dsdt=1−λs+b1pn+b2sn+b3sq+b4pq−s,

(41) dsndt=θ1λs−αnsn,

(42) dsqdt=1−θ1λs−αqsq,

(43) dpndt=βnsn−pn,

(44) dpqdt=βqsn+a1sq−pq,

(45) dcndt=γnpn−cn,

(46) dcqdt=γqpn+a2pq−cq,

(47) dindt=δncn−in,

(48) diqdt=δqcn+a3in+a4cq−iq,

(49) drrdt=cn+a5in+a6cq+a7iq−rr,

(50) drddt=μdin+a8iq−rd,

(51) drndt=hl,

(52) ddDdt=dD,

and the total populations satisfy the scaled equation

(53) dhldt=1−hl−b5in−b6iq,

(54) dhdt=1−h−b8rd,

where *b*_8_ > 0 if it is assumed that the rate of disposal of Ebola Virus Disease victims, *b*, is larger than the natural human death rate, *μ*. The scaled or dimensionless parameters are then as follows:

(55) ρn=θ3θ2aρNαNβN+μμ,ρq=θ7θ2bρQαNβQ+μμ,τn=θ3aθ2aτNαNβNβN+μrEN+γN+μμ,τq=θ3bθ2aτQαNβNβN+μrEQ+γQ+μμ,ξn=θ3aθ2aθ4ξNγNβNαNβN+μrEN+γN+μrLN+δN+μμ,ξq=θ3aθ2a1−θ4ξQγNβNαNβN+μrEN+γN+μrLQ+δQ+μμ,ad=θ2aθ3aθ4δNγNβNαNaDβN+μrEN+γN+μrLN+δN+μbμ,b1=1−θ3θ2aβNαNβN+μμ,b2=1−θ2αNμ,b3=1−θ6αQμ,b4=1−θ7θ2bβQαNβQ+μμ,b5=θ4θ2aθ3aδNγNβNαNrLN+δN+μrEN+γN+μβN+μμ,b6=1−θ4θ2aθ3aδQγNβNαNrLQ+δQ+μrEN+γN+μβN+μμ,b8=b−μθ4θ2aθ3aαNβNγNδNbμβN+μrEN+γN+μrLN+δN+μ,δn=rLN+δN+μμ,αn=αN+μμ,αq=αQ+μμ,βn=βN+μμ,βq=βQ+μμ,γn=rEN+γN+μμ,γq=rEQ+γQ+μμ,δq=rLQ+δQ+μμ,a1=θ6αQθ2bαN,a2=θ7θ2bβQβN+μθ2aθ3bβQ+μβN,a3=θ4σN1−θ4rLN+δN+μ,a4=θ3bγQrEN+γN+μ1−θ4θ3aγNrEQ+γQ+μ,a5=rLNθ4γNrENrLN+δN+μ,μd=bμ,a6=θ3brEQrEN+γN+μθ3arENrEQ+γQ+μ,a7=rLQ1−θ4γNrENrLQ+δQ+μ,a8=1−θ4δQrLN+δN+μθ4δNrLQ+δQ+μ.

### 3.3. The Steady State Solutions and Linear Stability

The steady state of the system is obtained by setting the right-hand side of the scaled system to zero and solving for the scalar equations. Let **x** ^*∗*^ = (*s* ^*∗*^, *s*_*n*_^*∗*^, *s*_*q*_^*∗*^, *p*_*n*_^*∗*^, *p*_*q*_^*∗*^, *c*_*n*_^*∗*^, *c*_*q*_^*∗*^, *i*_*n*_^*∗*^, *i*_*q*_^*∗*^, *r*_*r*_^*∗*^, *r*_*d*_^*∗*^, *h* ^*∗*^) be a steady state solution of the system. Then, (43), (45), and (47) indicate that

(56) $$\begin{array}{c} s_{n}^{*}=p_{n}^{*}=c_{n}^{*}=i_{n}^{*} \end{array}$$

and we can use any of these as a parameter to derive the values of the other steady state variables. We use the variables *p*_*n*_^*∗*^ and *s*_*q*_^*∗*^ as parameters to obtain the expressions

(57) pq∗pn∗,sq∗=pn∗+a1sq∗,cq∗pn∗,sq∗=A1pn∗+A2sq∗,iq∗pn∗,sq∗=A3pn∗+A4sq∗,rr∗pn∗,sq∗=A5pn∗+A6sq∗,rd∗pn∗,sq∗=A7pn∗+A8sq∗,h∗pn∗,sq∗=1−B1pn∗−B2sq∗,s∗pn∗,sq∗=1−B3pn∗−B4sq∗,hl∗pn∗,sq∗=1−b5+b6A3pn∗−b6A4sq∗,

where

(58) A1=1+a2,A2=a1a2,A3=1+a3+a4A1,A4=a4A2,A5=1+a5+a6A1+a7A3,A6=a6A2+a7A4,A7=1+a8A3,A8=a8A4,B1=b8A7,B2=b8A8,B3=αn−b1−b2−b4,B4=αq−b3−b4a1.

Here the solution for the scaled total living (*h*_*l*_) and scaled living and Ebola-deceased (*h*) populations is, respectively, obtained by equating the right-hand sides of (53) and (54) to zero, while that for *s* ^*∗*^ is obtained by adding up (40), (41), and (42). It is easy to verify from reparameterisation (38) that the parameter groupings *B*_3_ and *B*_4_ are both nonnegative. In fact,

(59) B31+αNμβNθ2aθ3−1βN+μ+βQθ2bθ7−1βQ+μ+θ2>0since  θ2=θ2a+θ2b,B4αQθ6βQθ7+μ+μβQ+μμβQ+μ>0,

showing that *B*_3_ > 0 and *B*_4_ > 0.

To obtain a value for *p*_*n*_^*∗*^ and *s*_*q*_^*∗*^, we substitute all computed steady state values, (56) and (57), into (41) and (42). The expression for *λ* ^*∗*^ *s* ^*∗*^ in terms of *p*_*n*_^*∗*^ and *s*_*q*_^*∗*^ is obtained from (39). Performing the aforementioned procedures leads to the two equations

(60) θ1B5pn∗+B6sq∗1−B3pn∗−B4sq∗=αnpn∗1−B1pn∗−B2sq∗,

(61) 1−θ1B5pn∗+B6sq∗1−B3pn∗−B4sq∗=αqsq∗1−B1pn∗−B2sq∗,

where

(62) B5=ρn+ρq+τn+τqA1+ξn+ξqA3+adA7,B6=ρqa1+τqA2+ξqA4+adA8.

Next, we solve (60) and (61) simultaneously, which clearly differ in some of their coefficients, to obtain the expressions for *p*_*n*_^*∗*^ and *s*_*q*_^*∗*^. Quickly observe that the two equations may be reduced to one such that

(63) $$\begin{array}{c} \left(\;1-B_{1}p_{n}^{*}-B_{2}s_{q}^{*}\;\right)\left(\;\alpha_{n}p_{n}^{*}\left(\;1-\theta_{1}\;\right)-\alpha_{q}s_{q}^{*}\theta_{1}\;\right)=0. \end{array}$$

Two possibilities arise: either (i) 1 − *B*_1_ *p*_*n*_^*∗*^ − *B*_2_ *s*_*q*_^*∗*^ = 0 or (ii) *α*_*n*_ *p*_*n*_^*∗*^(1 − *θ*_1_) − *α*_*q*_ *s*_*q*_^*∗*^ *θ*_1_ = 0. The first condition leads to the system

(64) 1−B3pn∗−B4sq∗=0,1−B1pn∗−B2sq∗=0.

However, the two equations are equivalent to *h* ^*∗*^ = 0 and *s* ^*∗*^ = 0 (see (57)), which are unrealistic, based on our constant population recruitment model. Hence, we only consider the second possibility, which yields the relation

(65) $$\begin{array}{c} p_{n}^{*}=\left(\;\frac{\alpha_{q}\theta_{1}}{\alpha_{n}\left(1-\theta_{1}\right)}\;\right)s_{q}^{*}, \end{array}$$

so that substituting (65) into (60) yields

(66) sq∗0,or  sq∗B7B8=1−θ1αnR0−1θ1αqB1+1−θ1αnB2R−1=xR0−1R−1,

where

(67) B7=αqαn1−θ1θ1B5αn+1−θ1B6αq−1=αqαn1−θ1R0−1,B8=αqθ1B5αn+1−θ1B6αq·αqθ1B3+αn1−θ1B4−θ1B1αq+αn1−θ1B2=αqθ1B1αq+αn1−θ1B2θ1B5αn+1−θ1B6αq·αqθ1B3+αn1−θ1B4θ1B1αq+αn1−θ1B2−1=αqθ1B1αq+αn1−θ1B2R0z−1,

with

(68) x=1−θ1αnθ1αqB1+1−θ1αnB2,y=θ1αqx1−θ1αn,z=θ1αqB3+1−θ1αnB4θ1αqB1+1−θ1αnB2,

(69) R0=θ1B5αn+1−θ1B6αq,R=R0θ1αqB3+1−θ1αnB4θ1αqB1+1−θ1αnB2=zR0.

**Remark 7 .**

It can be shown that *B*_2_ < *B*_4_. In fact, 

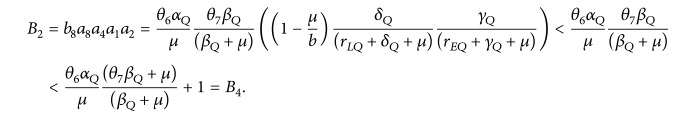

(70)

Thus, if (1 − *θ*_1_)*α*_*n*_(*B*_4_ − *B*_2_) > *θ*_1_ *α*_*q*_(*B*_1_ − *B*_3_), then *z* > 1. This will hold if *B*_3_ > *B*_1_. In the case where *B*_3_ < *B*_1_, we will require that *B*_4_ − *B*_2_ be greater than (*θ*_1_ *α*_*q*_/(1 − *θ*_1_)*α*_*n*_)(*B*_1_ − *B*_3_).

We identify *R*_0_ as the unique threshold parameter of the system as follows.

**Lemma 8 .**

The parameter *R*_0_ defined in (69) is the unique threshold parameter of the system whenever *z* > 1.

**Proof**

If *z* > 1, then *ℛ* = *zR*_0_ > 1 whenever *R*_0_ > 1 and the existence or nonexistence of a realistic solution of the form of (66) is determined solely by the size of *R*_0_.

The rest of the steady states are then obtained by using these values for *p*_*n*_^*∗*^ and *s*_*q*_^*∗*^ given by (66) in (65) and (57) to obtain the following:

(71) sq∗=xR0−1R−1,in∗=cn∗=sn∗=pn∗=yR0−1R−1,pq∗=y+a1xR0−1R−1,cq∗=A1y+A2xR0−1R−1,iq∗=A3y+A4xR0−1R−1,rr∗=A5y+A6xR0−1R−1,rd∗=A7y+A8xR0−1R−1,s∗=1−B3y+B4xR0−1R−1,h∗=1−B1y+B2xR0−1R−1,

where *x*, *y*, and *z* are as defined in (68). We have proved the following result.

**Theorem 9 (on the existence of equilibrium solutions).**

System (40)–(52) has at least two equilibrium solutions: the disease-free equilibrium **x** ^*∗*^ = *E*_*dfe*_ = (1, 0, 0, 0, 0, 0, 0, 0, 0, 0, 0, 1) and an endemic equilibrium **x** ^*∗*^ = *E*_*ee*_ = (*s* ^*∗*^, *s*_*n*_^*∗*^, *s*_*q*_^*∗*^, *p*_*n*_^*∗*^, *p*_*q*_^*∗*^, *c*_*n*_^*∗*^, *c*_*q*_^*∗*^, *i*_*n*_^*∗*^, *i*_*q*_^*∗*^, *r*_*r*_^*∗*^, *r*_*d*_^*∗*^, *h* ^*∗*^). The endemic equilibrium, *E*_*ee*_, exists and is realistic only when the threshold parameters *R*_0_ and *ℛ*, given by (69), are of appropriate magnitude.

The stability of the steady states is governed by the sign of the eigenvalues of the linearizing matrix near the steady state solutions. If *J*(**x** ^*∗*^) is the Jacobian matrix at the steady state **x** ^*∗*^, then we have

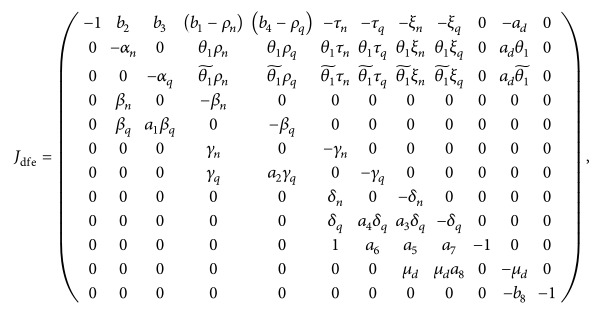

(72)
where $\tilde{\theta_{1}}=1-\theta_{1}$ . Thus if *ζ* is an eigenvalue of the linearized system at the disease-free state, then *ζ* is obtained by the solvability condition

(73) $$\begin{array}{c} P\left(\;\zeta \;\right)=\left|\;J_{dfe}-\zeta I\;\right|={\left(\;\zeta +1\;\right)}^{3}P_{9}\left(\;\zeta \;\right)=0, \end{array}$$

an equation involving a polynomial of degree 12 in *ζ*, where *P*_9_(*ζ*) is a polynomial of degree 9 in *ζ*, given by

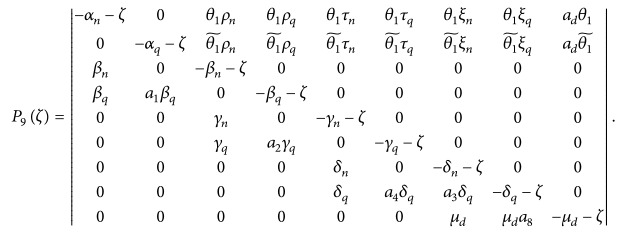

(74)
Now, all we need to know at this stage is whether there is solution of (73) for *ζ* with positive real part which will then indicate the existence of unstable perturbations in the linear regime. The coefficients of polynomial (73) can give us vital information about the stability or instability of the disease-free equilibrium. For example, by Descartes' rule of signs, a sign change in the sequence of coefficients indicates the presence of a positive real root which in the linear regime signifies the presence of exponentially growing perturbations. We can write polynomial equation (73) in the form

(75) $$\begin{array}{c} P\left(\;\zeta \;\right)={\left(\;\zeta +1\;\right)}^{3}\overset{9}{\underset{k=0}{\sum }}c_{i}\zeta^{i}, \end{array}$$

where

(76) c9=1,c8=αn+αq+βn+βq+γn+γq+δn+δq+μd,⋮c0=βnβqγnγqδnδqμdαnαq1−θ1B5αn−1−θ1B6αq=βnβqγnγqδnδqμdαnαq1−R0,

and we can see that *c*_0_ changes sign from positive to negative when *R*_0_ increases from values of *R*_0_ < 1 through *R*_0_ = 1 to values of *R*_0_ > 1 indicating a change in stability of the disease-free equilibrium as *R*_0_ increases from unity.

### 3.4. The Basic Reproduction Number

A threshold parameter that is of essential importance to infectious disease transmission is the basic reproduction number denoted by *R*_0_. *R*_0_ measures the average number of secondary clinical cases of infection generated in an absolutely susceptible population by a single infectious individual throughout the period within which the individual is infectious [26–29]. Generally, the disease eventually disappears from the community if *R*_0_ < 1 (and in some situations there is the occurrence of backward bifurcation) and may possibly establish itself within the community if *R*_0_ > 1. The critical case *R*_0_ = 1 represents the situation in which the disease reproduces itself thereby leaving the community with a similar number of infection cases at any time. The definition of *R*_0_ specifically requires that initially everybody but the infectious individual in the population be susceptible. Thus, this definition breaks down within a population in which some of the individuals are already infected or immune to the disease under consideration. In such a case, the notion of reproduction number *ℜ* becomes useful. Unlike *R*_0_ which is fixed, *ℜ* may vary considerably with disease progression. However, *ℜ* is bounded from above by *R*_0_ and it is computed at different points depending on the number of infected or immune cases in the population.

One way of calculating *R*_0_ is to determine a threshold condition for which endemic steady state solutions to the system under study exist (as we did to derive (69)) or for which the disease-free steady state is unstable. Another method is the next-generation approach where *R*_0_ is the spectral radius of the next-generation matrix [26]. Using the next-generation approach, we identify all state variables for the infection process, *p*_*n*_, *p*_*q*_, *c*_*n*_, *c*_*q*_, *i*_*n*_, *i*_*q*_, *r*_*r*_, *r*_*d*_, and *h*. The transitions from *s*_*n*_, *s*_*q*_ to *p*_*n*_, *p*_*q*_ are not considered new infections but rather a progression of the infected individuals through the different stages of disease compartments. Hence, we identify terms representing new infections from the above equations and rewrite the system as the difference of two vectors $\tilde{𝔉}$ and $\tilde{𝒱}$ , where $\tilde{𝔉}$ consists of all new infections and $\tilde{𝒱}$ consists of the remaining terms or transitions between states. That is, we set $\dot{x}=\tilde{𝔉}-\tilde{𝒱}$ , where **x** is the vector of state variables corresponding to new infections: **x** = (*s*, *s*_*n*_, *s*_*q*_, *p*_*n*_, *p*_*q*_, *c*_*n*_, *c*_*q*_, *i*_*n*_, *i*_*q*_, *r*_*r*_, *r*_*d*_, *h*)^*T*^. This gives rise to

(77) F~=0θ1λs1−θ1λs000000000,V~=−1+λs−b1pn−b2sn−b3sq−b4pq+sαnsnαqsqβn−sn+pnβq−sn−a1sq+pqγn−pn+cnγq−pn−a2pq+cqδn−cn+inδq−cn−a3in−a4cq+iq−cn−a5in−a6cq−a7iq+rr−μdin−μda8iq+μdrd−1+h+b8rd,

where the force of infection *λ* is given by (39). To obtain the next-generation operator, *FV* ^−1^, we must calculate $(F{)}_{ij}=\partial {\tilde{𝔉}}_{i}/\partial x_{j}$ and $(V{)}_{ij}=\partial {\tilde{𝒱}}_{i}/\partial x_{j}$ evaluated at the disease-free equilibrium position, where *s* = 1 = *h*, *s*_*n*_ = *s*_*q*_ = *p*_*n*_ = *p*_*q*_ = *c*_*n*_ = *c*_*q*_ = *i*_*n*_ = *i*_*q*_ = *r*_*r*_ = *r*_*d*_ = 0. The basic reproduction number is then the spectral radius of the next-generation matrix *FV* ^−1^. Thus if *ϱ*(*FV* ^−1^) is the spectral radius of the matrix *FV* ^−1^, then

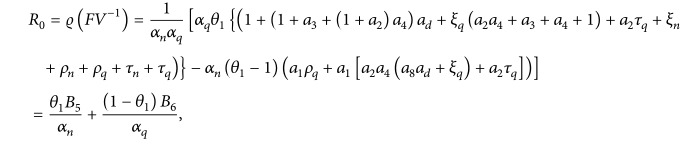

(78)
as computed before.

The expression for *R*_0_ has two parts. The first part measures the number of new EVD cases generated by an infected nonquarantined human. It is the product of *θ*_1_ (the proportion of susceptible individuals who become suspected but remain nonquarantined), *B*_5_ (which indicates the contacts from this proportion of individuals with infected individuals at various stages of the disease), and 1/*α*_*n*_ (which is the average duration a human remains as a suspected nonquarantined individual). In the same way, the second part can be interpreted likewise.

The stability of the endemic steady state is obtained by calculating the eigenvalues of the linearized matrix evaluated at the endemic state. The computations soon become very complicated because of the size of the system and we proceed with a simplification of the system.

### 3.5. Pseudo-Steady State Approximation

Ebola Virus Disease is a very deadly infection that normally kills most of its victims within about 21 days of exposure to the infection. Thus when compared with the life span of the human, the elapsed time representing the progression of the infection from first exposure to death is short when compared to the total time required as the life span of the human. Thus we set *μ* ≈ 1/life  span  of  human, so that the rates *α*_*∗*_, *β*_*∗*_, and so forth will all be such that 1/rate ≈ resident  time  in  given  state, some of which will be short compared with the life span of the human. It is therefore reasonable to assume that

(79) $$\begin{array}{c} \frac{\mu }{\mu +rate}\approx small⟹\frac{\mu +rate}{\mu }\approx large, \end{array}$$

so that the scaling above renders some of the state variables essentially at equilibrium. That is, the quantities, 1/*β*_*n*_, 1/*β*_*q*_, 1/*γ*_*n*_, 1/*γ*_*q*_, 1/*δ*_*n*_, 1/*δ*_*q*_, and *b*/*μ*, may be regarded as small parameters so that, in the corresponding equations (43)–(48) and (50), the state variables modelled by these equations are essentially in equilibrium and we can evoke the Michaelis-Menten pseudo-steady state hypothesis [30]. To proceed, we make the pseudoequilibrium approximation

(80) pn=cn=in=sn,pq=sn+a1sq,cq=A1sn+A2sq,iq=A3sn+A4sq,rd=A7sn+A8sq

to have the reduced system

(81) dsdt=1−λs+αn−B3sn+αq−B4sq−s,

(82) dsndt=θ1λs−αnsn,

(83) dsqdt=1−θ1λs−αqsq,

(84) drrdt=A5sn+A6sq−rr,

(85) dhdt=1−h−B1sn−B2sq,

and the total population and the force of infection also reduce accordingly. In particular, we have

(86) λs=ρnpnh+ρqpqh+τncnh+τqcqh+ξninh+ξqiqh+adrdhs=B5snh+B6sqhs,

where the variables *s* and the augmented population *h* satisfy the differential equations (81) and (85), respectively.

System (81)–(85) has the same steady states solutions as the original system if we combine it with (80). However, on its own, it represents a pseudo-steady state approximation [30] of the original system. Clearly the reduced system has two realistic steady states: *E*_dfe_ and *E*_*ee*_, so that if *E*_**x** ^*∗*^_ = (*s* ^*∗*^, *s*_*n*_^*∗*^, *s*_*q*_^*∗*^, *r*_*r*_^*∗*^, *h* ^*∗*^) is a steady state solution, then

(87) Edfe=1,0,0,0,1,Eee=s∗,sn∗,sq∗,rr∗,h∗,

where, following the same method as was done in the full system,

(88) sq∗=B7B8,sn∗=αqθ1αn1−θ1sq∗,rr∗=A5sn∗+A6sq∗,s∗=1−B3sn∗−B4sq∗,h∗=1−B1sn∗−B2sq∗.

All coefficients are as defined in (58). When steady states (88) are rendered in parameters of the reduced system, taking into consideration the fact that, from (68), *z* = *B*_3_ *y* + *B*_4_ *x* and *B*_1_ *y* + *B*_2_ *x* = 1, we get

(89) s∗=z−1R−1,sn∗=yR0−1R−1,sq∗=xR0−1R−1,rr∗=A5y+A6xR0−1R−1,h∗=z−1R0R−1.

The stability of the steady states is determined by the eigenvalues of the linearized matrix of the reduced system evaluated at the steady state **x** ^*∗*^ = (*s* ^*∗*^, *s*_*n*_^*∗*^, *s*_*q*_^*∗*^, *r*_*r*_^*∗*^, *h* ^*∗*^). If *J*(**x** ^*∗*^) is the Jacobian of the system near the steady state **x** ^*∗*^, then

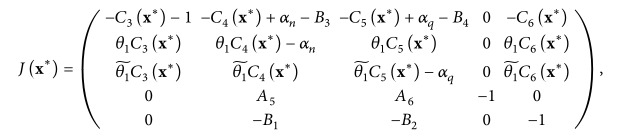

(90)
where $\tilde{\theta_{1}}=1-\theta_{1}$ and

(91) C3x∗=B5sn∗h∗+B6sq∗h∗=αnyR0−1θ1z−1,C4x∗=B5s∗h∗=B5R0,C5x∗=B6s∗h∗=B6R0,C6x∗=−B5s∗sn∗h∗2+B6s∗sq∗h∗2=−αnyR0−1θ1z−1R0.

The asterisk is used to indicate that the quantities so calculated are evaluated at the steady state. We can perform a stability analysis on the reduced system by noting that if *ζ* is an eigenvalue of (90), then *ζ* satisfies the polynomial equation

(92) P5ζ,x∗=ζ+12ζ3+Q2x∗ζ2+Q1x∗ζ+Q0x∗=0,

where

(93) Q2x∗=αn+αq+C3x∗−C4x∗θ1−C5x∗θ1~+1,Q1x∗=αq−θ1−B1C6x∗+αqC4x∗+C4x∗+B2C6x∗θ1~+C3x∗αqθ1+B3θ1+B4θ1~+αnαq+θ1~C3x∗−C5x∗θ1~+1−C5x∗·θ1~,Q0x∗=αqθ1B1C6x∗+B3C3x∗−C4x∗+αnθ1~B2C6x∗+B4C3x∗−C5x∗+αq.

Now, the signs of the zeros of (92) will depend on the signs of the coefficients *Q*_*i*_, *i* ∈ {0,1, 2}. We now examine these.

At the disease-free state where *s* ^*∗*^ = 1 = *h* ^*∗*^, *s*_*n*_^*∗*^ = *s*_*q*_^*∗*^ = *r*_*r*_^*∗*^ = 0, or equivalently *R*_0_ = 1, we have **x** ^*∗*^ = **x**_dfe_ = (1,0, 0,0, 1) so that *C*_3_(**x**_dfe_) = 0, *C*_4_(**x**_dfe_) = *B*_5_, *C*_5_(**x**_dfe_) = *B*_6_, *C*_6_(**x**_dfe_) = 0, and (92) becomes

(94) $$\begin{array}{c} P_{5}\left(\;\zeta ,x_{dfe}\;\right)={\left(\;\zeta +1\;\right)}^{3}\left(\;\zeta^{2}+Q_{dfe}\zeta +R_{dfe}\;\right)=0, \end{array}$$

where

(95) Qdfeαn+αq−B5θ1−B61−θ1=αn1−R0+αq+1−θ1B6αn−αqαq,Rdfeαqαn−B5θ1+αnB6θ1−1=αqαn1−R0.

The roots of (94) are −1, −1, −1, and $(-Q_{dfe}\pm \sqrt{Q_{dfe}^{2}-4\alpha_{q}\alpha_{n}(1-R_{0})})/2$ , showing that there is one positive real solution as *R*_0_ increases beyond unity and the disease-free equilibrium loses stability at *R*_0_ = 1. For the local stability when *R*_0_ ≤ 1, the additional requirement *Q*_dfe_ > 0 is necessary.

At the endemic steady state, and in the original scaled parameter groupings of the system, **x** ^*∗*^ = **x**_*ee*_ = (*s* ^*∗*^, *s*_*n*_^*∗*^, *s*_*q*_^*∗*^, *r*_*r*_^*∗*^, *h* ^*∗*^), the coefficients of (92) simplify accordingly and we have

(96) $$\begin{array}{c} P_{5}\left(\;\zeta ,x^{*}\;\right)={\left(\;\zeta +1\;\right)}^{2}\left(\;\zeta^{3}+P_{x_{ee}}\zeta^{2}+Q_{x_{ee}}\zeta +R_{x_{ee}}\;\right)=0, \end{array}$$

where

(97) Pxee=B6θ1θ1~z−1αn−αq+αqR0αnR0−1y+αq+1θ1z−1αqθ1R0z−1,Qxee=αnθ1~Q11+αq2R0Q12αnαqR0z−1θ1~,Rxee=R0−1R−1αnαqz−1R0,

where

(98) Q11=B6z−1θ1~αq−αn+αqαqB2x+R02z+αnR0−1B2x+αqR0x−1,Q12=αn−θ1B2x+R0−1xαq+B3+1+B2x+1+αqB3θ1R0−1x.

Now the necessary and sufficient conditions that will guarantee the stability of the nontrivial steady state **x**_*ee*_ will be the Routh-Hurwitz criteria which, in the present parameterizations, are

(99) Pxee>0,Qxee>0,Rxee>0,PxeeQxee−Rxee>0.

With this characterization, we can then explore special cases of intervention.

### 3.6. Some Special Cases

**Figure 1:**
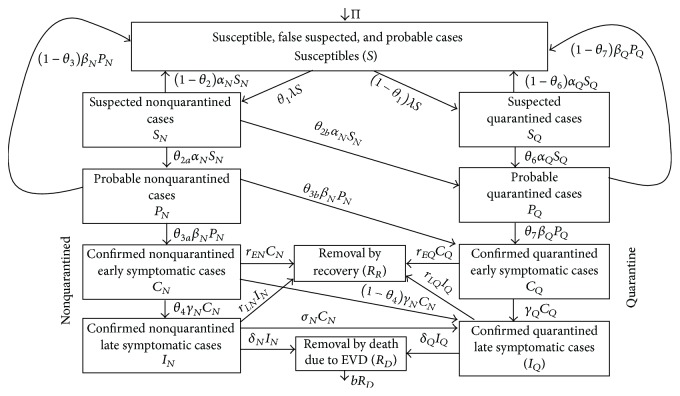
Conceptual framework showing the relationships between the different compartments that make up the different population of individuals and actors in the case of an EVD outbreak. Susceptible individuals include false suspected and probable cases. True suspects and probable cases are confirmed by a laboratory test and the confirmed cases can later develop symptoms and die of the infection or recover to become immune to the infection. Humans can also die naturally or due to other causes. Nonquarantined cases can become quarantined through intervention strategies. Others run the course of the illness from infection to death without being quarantined. Flow from compartment to compartment is as explained in the text.

All initial suspected cases are quarantined: that is, *θ*_1_ = 0. In this case we see that *s*_*n*_ = 0 and we have only the right branch of our flow chart in Figure 1. We have here a problem involving infections only at the treatment centres. Mathematically, we then have

(100) R0=B6αq,y=0,x=1B2,z=B4B2,C3=αqxR0−1z−1,C6=−C3R0

and (96) becomes

(101) P5ζ,x∗=ζ+αnζ+12ζ2+1+R0−1xαqz−1ζ+R−1R0−1αqR0z−1,

showing that all solutions of the equation *P*_5_(*ζ*, **x** ^*∗*^) = 0 are negative or have negative real parts whenever they are complex, indicating that the nontrivial steady state is stable to small perturbations whenever *R*_0_ > 1. In this case we can regard an increase in *R*_0_ as an increase in the parameter grouping *B*_6_.

All initial suspected cases escape quarantine: that is, *θ*_1_ = 1. In this case we see that *s*_*q*_ = 0 and initially we will be on the left branch of our flow chart in Figure 1. The strength of the present model is that, based on its derivation, it is possible for some individuals to eventually enter quarantine as the systems wake up from sleep and control measures kick into place. Mathematically, we then have

(102) R0=B5αn,x=0,y=1B1,z=B3B1,C3=αnyR0−1z−1,C6=−C3R0

and (96) becomes

(103) P5ζ,x∗=ζ+αnζ+12ζ2+1+R0−1yαnz−1ζ+R−1R0−1αnR0z−1,

again showing that all solutions of the equation *P*_5_(*ζ*, **x** ^*∗*^) = 0 are negative or have negative real parts whenever they are complex, indicating that the nontrivial steady state is stable to small perturbations whenever *R*_0_ > 1. In this case we can regard an increase in *R*_0_ as an increase in the parameter grouping *B*_5_. *R*_0_ in this case appears larger than in the previous cases.

The rate at which suspected individuals become probable cases is the same: that is, *α*_*N*_ = *α*_*Q*_. In this case the flow from being a suspected case to a probable case is the same in all circumstances, irrespective of whether or not one is quarantined or nonquarantined. Mathematically, we have that *α*_*n*_ = *α*_*q*_ and (96) becomes

(104) P5ζ,x∗=ζ+αqζ+12ζ2+1+R0−1xαqz−11−θ1ζ+R−1R0−1αqR0z−1.

In this case as well, all solutions of the equation *P*_5_(*ζ*, **x** ^*∗*^) = 0 either are negative or have negative real parts whenever *R*_0_ > 1 and *z* > 1, showing that in this case again the steady state is stable to small perturbations. In this particular case, an increase in *R*_0_ can be regarded as an increase in the two parameter groupings *B*_5_ and *B*_6_.

## 4. Parameter Discussion

Some parameter values were chosen based on estimates in [15, 20], on the 2014 Ebola outbreak, while others were selected from past estimates (see [9, 14, 18]) and are summarized in Table 2. In [20], an estimate based on data primarily from March to August 20th yielded the following average transmission rates and 95% confidence intervals: 0.27 (0.27,0.27) per day in Guinea, 0.45 (0.43,0.48) per day in Sierra Leone, and 0.28 (0.28,0.29) per day in Liberia. In [15], the number of cases of the 2014 Ebola outbreak data (up until early October) was fitted to a discrete mathematical model, yielding estimates for the contact rates (per day) in the community and hospital (considered quarantined) settings as 0.128 in Sierra Leone for nonquarantined cases (and 0.080 for quarantined cases, about a 61% reduction) while the rates in Liberia were 0.160 for nonquarantined cases (and 0.062 for quarantined cases, close to a 37.5% drop). The models in [15, 20] did not separate transmission based on early or late symptomatic EVD cases, which was considered in our model. Based on the information, we will assume that effective contacts between susceptible humans and late symptomatic EVD patients in the communities fall in the range [0.12,0.48] per day, which contains the range cited in [16]. Thus *ξ*_*N*_ ∈ [0.12,0.48]. However, we will consider scenarios in which this parameter varies. Furthermore, if we assume about a 37.5% to 61% reduction in effective contact rates in the quarantined settings, then we can assume that *ξ*_*Q*_ = *ϕ*_*Q*_ *ξ*_*N*_, where *ϕ*_*Q*_ ∈ [0.0375,0.062]. However, to understand how effective quarantining Ebola patients is, general values of *ϕ*_*Q*_ ∈ [0,1] can be considered. Under these assumptions, a small value of *ϕ*_*Q*_ will indicate that quarantining was effective, while values of *ϕ*_*Q*_ close to 1 will indicate that quarantining patients had no effect in minimizing contacts and reducing transmissions.

Since patients with EVD at the onset of symptoms are less infectious than EVD patients in the later stages of symptoms [2, 10], we assume that the effective contact rate between confirmed nonquarantined early symptomatic individuals and susceptible individuals, denoted by *τ*_*N*_, is proportional to *ξ*_*N*_ with proportionality constant *q*_*N*_ ∈ [0,1]. Likewise, we assume that the effective contact rate between confirmed quarantined early symptomatic individuals and susceptible individuals is proportional to *ξ*_*q*_ with proportionality constant *q*_*q*_ ∈ [0,1]. Thus, *τ*_*N*_ = *q*_*N*_ *ξ*_*N*_ and *τ*_*Q*_ = *q*_*Q*_ *ξ*_*Q*_, with 0 < *q*_*N*_, *q*_*Q*_ < 1.

The range, for the parameter *a*_*D*_, of the effective contact rate between cadavers of confirmed late symptomatic individuals and susceptible individuals was chosen to be [0.111,0.489] per day, where 0.111 is the rate estimated in [15] for Sierra Leone and 0.489 is that for Liberia. With control measures and education in place, these rates can be much lower.

The incubation period of EVD is estimated to be between 2 and 21 days [2, 7–9], with a mean of 4–10 days reported in [8, 9]. In [10], a mean incubation period of 9–11 days was reported for the 2014 EVD. Here, we will consider a range from 4 to 11 days, with a mean of about 10 days used as the baseline value. Thus we will consider that *α*_*N*_ and *α*_*Q*_ are in the range [1/11,1/4]. At the end of the incubation period, early symptoms may emerge 1–3 days later [10], with a mean of 2 days. Thus the mean rate at which nonquarantined (*β*_*N*_) and quarantined (*β*_*Q*_) suspected cases become probable cases lies in [1/3,1] [12]. About 1 or 2 to 4 days later after the early symptoms, more severe symptoms may develop so that the rates at which nonquarantined (*γ*_*N*_) and quarantined (*γ*_*Q*_) probable cases become confirmed cases lie in [1/4,1/2].

The parameter *γ*_*N*_ measures the rate at which early symptomatic individuals leave that class. This could be as a result of recovery or due to increase and spread of the virus within the human. It takes about 2 to 4 days to progress from the early symptomatic stage to the late symptomatic stage, so that *γ*_*N*_, *γ*_*Q*_ ∈ [1/4,1/2], which can be assumed to be the reciprocal of the mean time it takes from when the immune system is either completely overwhelmed by the virus or kept in check via supportive mechanism. Severe symptoms are followed either by death after about an average of two to four days beyond entering the late symptomatic stage or by recovery [12], and thus we can assume that *δ*_*N*_ and *δ*_*Q*_ are in the range [1/4,1/2]. If on the other hand the EVD patient recovers, then it will take longer for patient to be completely clear of the virus. Notice that, based on the ranges given above, the time frames are [6, 16] days from the onset of symptoms of Ebola to death or recovery. The range from the onset of symptoms which commences the course of illness to death was given as 6–16 days in [8], while the range for recovery was cited as 6–11 days [8]. For our baseline parameters, the mean time from the onset of the illness to death or recovery will be in the range of 6–11 days.

Here, we will consider that the recovery rate for quarantined early symptomatic EVD patients lies in the range [0.4829,0.5903] per day with a baseline value of 0.5366 per day, as cited in [16]. Thus *r*_*EQ*_ ∈ [0.4829,0.5903]. If we assume that patients quarantined in the hospital have a better chance of surviving than those in the community or at home, without the necessary expert care that some of the quarantined EVD patients may get, then we can consider that the recovery rate for nonquarantined EVD patients, in the community, would be slightly lower. Thus we scale *r*_*EQ*_ by some proportion *ω* ∈ [0,1], so that *r*_*EN*_ = *ωr*_*EQ*_. Since late symptomatic patients have a much lower recovery chance, then both *r*_*LN*_ and *r*_*LQ*_ will be lower than *r*_*EN*_ and *r*_*EQ*_, respectively. Hence we will consider that *r*_*LN*_ = *κr*_*EN*_ and *r*_*LQ*_ = *κr*_*EQ*_, where 0 < *κ* ≪ 1. Sometimes late symptomatic EVD patients are removed from the community and quarantined. Here we assume a mean of 2 days so that *σ*_*N*_ = 0.5 per day.

The fractions *θ*_*i*_ measure the proportions of individuals moving into various compartments. If we assume that members in the quarantined classes are not left unchecked but have medical professionals checking them and giving them supportive remedies to boost their immune system to fight the Ebola Virus or enable their recovery, then it will be reasonable to assume that *θ*_6_, *θ*_7_, *θ*_4_, and *θ*_5_ are all greater than 0.5. If such an assumption is not made, then the parameters can be chosen to be equal or close to each other.

The parameter Π is chosen to be 555 per day as in [16]. Furthermore, the parameters *ρ*_*N*_ and *ρ*_*Q*_ and the effective contact rates between probable nonquarantined and, respectively, quarantined individuals and susceptible individuals will be varied to see their effects on the model dynamics. However, the values chosen will be such that the value of *R*_0_ computed is within realistic reported ranges.

The parameter *μ*, the natural death rate for humans, is chosen based on estimates from [13]. The parameter *b* measures the time it takes from death to burial of EVD patients. A mean value of 2 days was cited in [14] for the 1995 and 2000 Ebola outbreak epidemics in the Democratic Republic of Congo and Uganda, respectively. For the 2014 West African Outbreak, the estimates were 2.01 days in Liberia and 4.50 days in Sierra Leone [15].

## 5. Numerical Simulation of the Scaled Reduced Model

**Figure 2:**
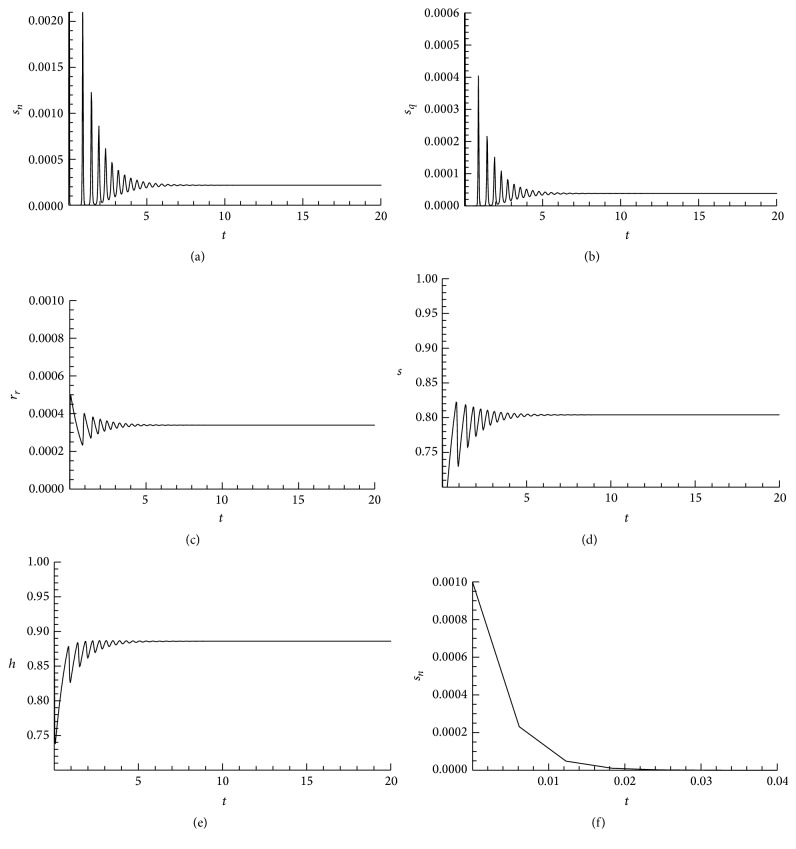
(a)–(e) Time series showing convergence of the solutions to the steady states for the nondimensional reduced model when the constant recruitment term is 555 persons per day. In this example, *θ*_1_ = 0.85 and *ρ*_*N*_ = 1.15 and *ρ*_*Q*_ = 0.85, and all the other parameters are as in Table 2, giving values of *R*_0_ = 1.102. In this case, the nonzero steady state is stable and the solution converges to the steady state value as given by (89) as *t* → *∞*. (f) Time series showing the long term behaviour of the variable *s*_*n*_ in the reduced model for *ρ*_*N*_ = *ρ*_*Q*_ = 0.8 and all other values of the parameters are as given in Table 2. In this case *R*_0_ = 0.88 and so the only steady state is the trivial steady state which is stable.

The parameter values given in Table 2 were used to carry out some numerical simulations for the reduced model, (81)–(85), when the constant recruitment term is 555 persons per day. The varying parameters were chosen so that *R*_0_ would be within ranges of reported values, which are typically less than 2.5 (see, e.g., [16, 22]). In Figure 2, we show a time series solution for a representative choice of values for the parameters *ρ*_*N*_ and *ρ*_*Q*_. Figures 2(a)–2(e) show the long term solutions to the reduced model exhibiting convergence to the stable nontrivial equilibrium in the case where *R*_0_ > 1, the case with sustained infection in the community.  Figure 2(f) then shows an example of convergence to the trivial steady state when *R*_0_ < 1, the case where the disease is eradicated. In that example we notice that as *R*_0_ < 1, *s*_*n*_ → 0 as *n* → *∞* and this in turn implies that *s*_*q*_ → 0 as *n* → *∞* and eventually the system relaxes to the trivial state (*s*, *s*_*n*_, *s*_*q*_, *r*_*r*_, *h*) = (1,0, 0,0, 1) for large time.

We note here that the computed value of *R*_0_ can be shown to be linear in the variables *ρ*_*Q*_ and *ρ*_*N*_, when eventually all parameters have been assigned; it may be written as *R*_0_ = *r*_0_ + *r*_1_ *ρ*_*Q*_ + *r*_2_ *ρ*_*N*_, where *r*_*i*_, *i* = 0,1, 2, are positive constants that can be shown to be dependent on the other parameters. Thus *R*_0_ will increase linearly with increase in any of the parameters *ρ*_*Q*_ and *ρ*_*N*_ for fixed given values of the other parameters. Though we have theoretically found, for example, that *s* ^*∗*^ becomes infinite when *ℛ* is near one equivalent to *R*_0_ being near 1/*z*, this case does not arise because we have assumed in the analysis that *z* > 1. Thus the case 0 ≤ *z* < 1 is linked with the trivial steady state.

**Figure 3 fig3:**
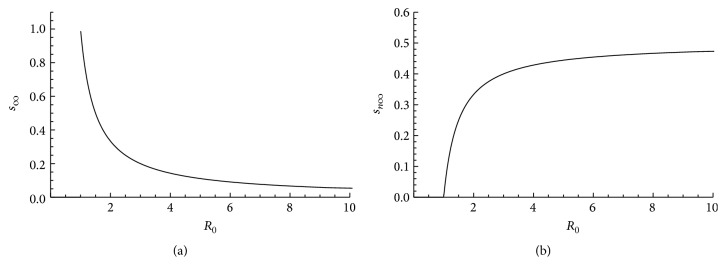
Graph showing the behaviour of the steady states *s* ^*∗*^ and *s*_*n*_^*∗*^ as a function of *R*_0_. (a) This graph shows the form of the steady state *s* ^*∗*^ as a function of *R*_0_. (b) This graph shows the form of the steady state *s*_*n*_^*∗*^ as a function of *R*_0_. The steady state solution *s*_*n*_^*∗*^ varies greatly only in a narrow band of reproduction numbers but saturates for large values of *R*_0_. On the other hand, *s* ^*∗*^ continues to drop to zero as *R*_0_ increases.

The situation shown in Figure 3 has important consequences for control strategies. While *s*_*n*_^*∗*^ varies sharply for a narrow band of reproduction numbers, its values do not change much for larger values of *R*_0_. Referring to Figure 3, an application of a control measure that will reduce *R*_0_ say from a high value of 10 down to 5, a 50% reduction, will not appreciably affect the rest of the disease transmission. Thus the system is best controlled when *R*_0_ is small which can occur in the early stages of the infection or late in the infection when some effective control measures have already been instituted such as effective quarantining or prompt removal of EVD deceased individuals. Notice that as *R*_0_ further increases, the number of susceptible individuals continues to drop. We note, however, that typical values of *R*_0_ computed for the 2014 Ebola outbreak are less than 2.5.

**Figure 4:**
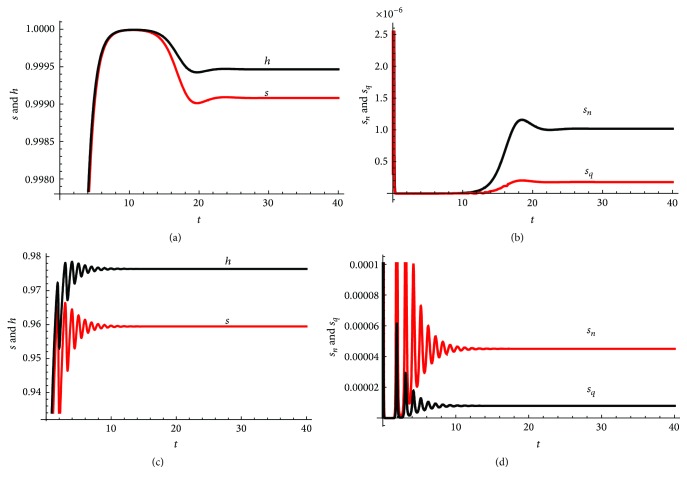
(a)–(d) Time series plot showing the propagation and stabilization of EVD to a stable nontrivial steady state for the nondimensional reduced model when the constant recruitment term is 555 persons per day and with *ρ*_*N*_ = *ρ*_*Q*_ = 0.8 and *θ*_1_ = 0.85 as used in Figure 2(f). Except for *ξ*_*N*_ that is increased from its baseline value of 0.27, all other parameters are as in Table 2. In graphs (a) and (b), *ξ*_*N*_ is increased to 0.453. This yields *R*_0_ = 1.00038, slightly bigger than 1. The graphs show that there is a major peak which starts to decay as EVD deaths begin to rise. The size of the epidemic can be estimated as the difference in the areas between the *s* and *h* curves as the disease settles to its steady state. Graphs (c) and (d) show the model dynamics when *ξ*_*N*_ is further increased to 0.48, which yields *R*_0_ = 1.01765. In graphs (c) and (d), the oscillations are more pronounced and the size of the epidemic is larger due to the increased effective contacts with late symptomatic individuals.

Next, we investigate the effect of *ξ*_*N*_ on *R*_0_ and the model dynamics. In Figure 2(f), we showed an example of convergence to the trivial steady state for *ρ*_*N*_ = 0.8 = *ρ*_*Q*_ for the nondimensional reduced model when the constant recruitment term is 555 persons per day. The model dynamics yielded an *R*_0_ value of 0.88 < 1, when all other parameters were as given in Table 2. From this scenario, we increased only *ξ*_*N*_ from its baseline Table 2 value of 0.27 to 0.453. This yields an increase in *R*_0_ to 1.00038 and we see from Figures 4(a) and 4(b) that the disease begins to propagate and stabilize within the community. There is a major peak which starts to decay as EVD deaths begin to rise. An estimated size of the epidemic can be computed as the difference between *s* and *h* when the disease dynamics settles to its equilibrium state. As more and more persons become infected, *R*_0_ increases and the estimated size of the epidemic also increases as is expected. In particular, increasing *ξ*_*N*_ further to 0.48 increases *R*_0_ to 1.01765, and, from Figures 4(c) and 4(d), the difference between *s* and *h* is visibly larger compared to the difference from Figures 4(a) and 4(b). Moreover, the oscillatory dynamics becoming more pronounced indicated a higher back and forth movement activity between the *s* and *s*_*n*_ and *s*_*h*_ classes.

**Figure 5:**
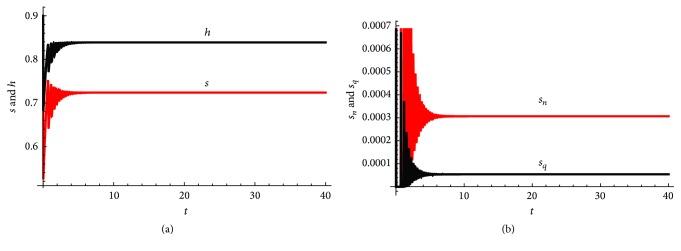
(a)-(b) Time series plot showing the propagation and stabilization of EVD to a stable nontrivial steady state for the nondimensional reduced model when the constant recruitment term is 555 persons per day and with *ρ*_*N*_ = 1.15, *ρ*_*Q*_ = 0.85, and *θ*_1_ = 0.85 as in Figures 2(a)–2(e). Except for *ξ*_*N*_ that is increased from 0.27 to 0.36, all the other parameters are as given in Table 2, and the corresponding *R*_0_ value is *R*_0_ = 1.159. Notice that, in this case, the disease has a higher frequency of oscillations and the difference between the areas under *h* and *s* is considerably larger indicating that the size of the disease burden is considerably larger in this case.

In Figures 2(a)–2(e), we showed an example of the long term dynamics of the solutions of the reduced model for *ρ*_*N*_ = 1.15 and *ρ*_*Q*_ = 0.85. For this case, we obtained *R*_0_ = 1.102 > 1 and the model dynamics show how the reduced model converges to a stable nontrivial equilibrium, when all other parameters are as stated as in Table 2. From this point, if we increase only *ξ*_*N*_ from its default value of 0.27 to 0.36, we see that *R*_0_ increases to approximately 1.159 and the model exhibits irregular random oscillations with higher frequency but eventually stabilizes (Figures 5(a) and 5(b)). The size of the epidemic is considerably larger in this case. This highlights the importance of reducing contacts between EVD patients and susceptible humans in controlling the size of the disease burden and lowering the impact of the disease.

### 5.1. Fade-Outs and Epidemics in Ebola Models

Our model results as highlighted in Figures 2(a)–2(e) [Fig fig3], 4, and 5 indicate that it is possible to have a long term endemic situation for Ebola transmission, if conditions are right. In particular, in our model, for the case where we have a relatively large constant recruitment term of 555 persons per day and with available resources to sustain the quarantine efforts then as long as there are people in the community (nonquarantined) with the possibility to come in contact with infectious EVD fluids, then the disease can be sustained as long as *R*_0_ > 1 (Figures 2(a)–2(e), 4, and 5). Increasing control by reducing contacts early enough between suspected and probable individuals with susceptible individuals can bring down the size of *R*_0_ to a value <1 which eventually leads to the eradication of the disease. Thus control which includes quarantining has to be comprehensive and sustained until eradication is achieved.

**Figure 6:**
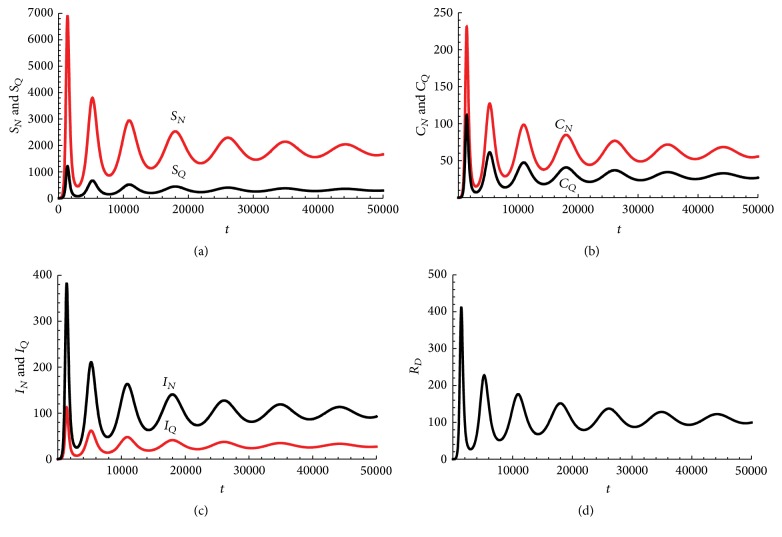
(a)–(c) Time series showing convergence of the solutions to the steady states for the full model in dimensional form when the constant recruitment term is 555 persons per day. In this example, *θ*_1_ = 0.85 and *ρ*_*N*_ = 1.15 and *ρ*_*Q*_ = 0.85, and all the other parameters are as in Table 2, giving a value of *R*_0_ = 1.102. The graph shows the short scale dynamics as well as the long term behaviour showing stability of the nonzero steady state.

The 2014 Ebola outbreak did not show sustained disease states. The disease dynamics exhibited epidemic fade-outs. Here, we show that such fade-outs are possible with our model. To investigate the epidemic-like fade-outs, we first note that due to the scaling adopted in our model, our time scales are large. However, any epidemic-like EVD behaviour would be expected to occur over a shorter timescale. Thus, for the results illustrated here, we plot the model dynamics in terms of the original variable by simulating the equations that make up the system, (11)–(23). To illustrate that the large time scales do not affect the long term dynamics of the model results, we first present a graph of the original system in the case where the parameters are maintained as those used in Figure 6 with Π = 555 persons per day, *θ*_1_ = 0.85 and *ρ*_*N*_ = 1.15 and *ρ*_*Q*_ = 0.85, and with all the other parameters as given in Table 2. The *R*_0_ value was 1.102 and so a sustained disease with no other effort is possible over a long time frame of more than 10,000 days.

**Figure 7:**
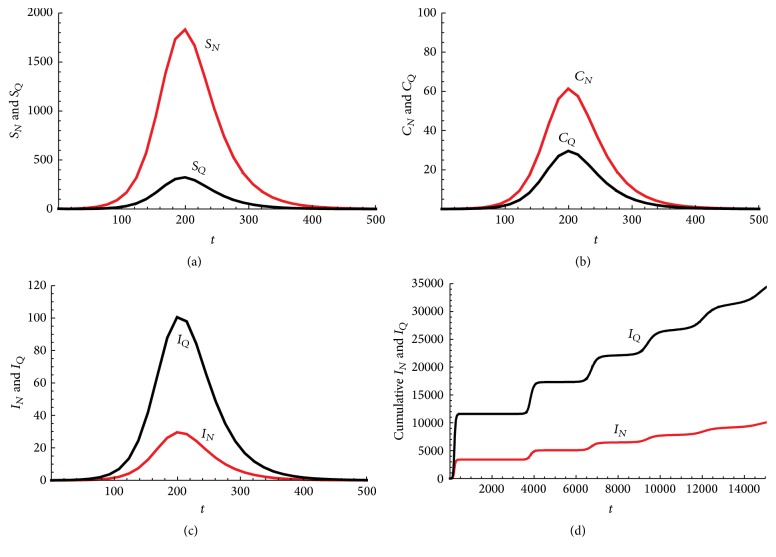
(a)-(b) Time series showing epidemic-like behaviours of the solutions to the steady states for the full model in dimensional form when the constant recruitment term is 3 persons per day over a short time scale. In this example, *θ*_1_ = 0.85 and *ρ*_*N*_ = 2 and *ρ*_*Q*_ = 1, and all the other parameters are as in Table 2. The value of *R*_0_ = 1.63455. The graph shows the short scale dynamics exhibiting epidemic-like behaviour that fades out.

When the number of individuals recruited daily reduces, then we can show that, for the case where Π = 3 persons per day, *θ*_1_ = 0.85 and *ρ*_*N*_ = 2 and *ρ*_*Q*_ = 1, and all the other parameters remain as in Table 2; then an epidemic-like behaviour is obtained (see Figure 7).

The dynamics of Figures 6 and 7 indicate that quarantining alone is not sufficient to eradicate the Ebola epidemic especially when there is a relative high number of daily recruitment. In fact, quarantining can instead serve as a buffer zone allowing the possibility of sustained disease dynamics when there are a reasonable number of people recruited each day. However, when the daily recruitment is controlled, reduced to a value of 3 per day, then the number of new daily infections is reduced to a low value as depicted in Figures 7(a)–7(c). However, the estimated cumulative number of infections increases daily (see Figure 7(d)).

## 6. Discussion and Conclusion

In this paper we set out to derive a comprehensive model for the dynamics of Ebola Virus Disease transmission in a complex environment where quarantining is not effective, meaning that some suspected cases escape quarantine while others do not. When the West African countries of Liberia, Guinea, and Sierra Leone came face to face with Ebola Virus Disease infection in 2014, it took the international community some time to react to the crises. As a result, most of the initial cases of EBV infection escaped monitoring and entered the community. African belief systems and other traditional practices further compounded the situation and before long large number of cases of EBV infections were in the community. Even when the international community reacted and started putting in place treatment centres, it still took some time for people to be sensitized on the dangers they are facing. The consequence was that infections continued in families, during funerals, and even in hospitals. People checked into hospitals and would not tell the truth about their case histories and as a result some medical practitioners got exposed to the infection. A case in point is that of Dr. Stella Ameyo Adadevoh, an Ebola victim and everyday hero [31], who prevented the spread of Ebola in Nigeria and paid with her life. We still pay tribute and honour to her and the other health workers whose dedication was inspirational and helpful in curbing the 2014 Ebola outbreak in Africa.

As we now look forward with optimism for a better and Ebola-free tomorrow, there is work going on in the scientific community to develop vaccines [2]. Mathematical modelling of the dynamics and transmission of Ebola provides unique avenues for exploration of possible management scenarios in the event of an EVD outbreak, since, during an outbreak, management of the cases is crucial for containment of the spread of the infection within the community. In this paper we have presented a comprehensive ordinary differential equation model that handles management issues of EVD infection. Our model takes care of quarantine and nonquarantine cases and therefore can be used to predict progression of disease dynamics in the population. Our analysis has shown that the initial response to all suspected cases of EVD infection is crucial. This is captured through the parameter *θ*_1_ which measures the initial fraction of suspected cases that are put into quarantine. We have shown that the basic reproduction number can be indexed by this parameter in the sense that when all cases are initially quarantined the spread of the infection can only take place at the treatment centres, but in cases where all suspected cases escape quarantine, the reproduction number can be large. Our model has been able to quantify the densities of infected and recovered individuals within the population based on baseline parameters identified during the 2014 Ebola Virus Disease outbreak in Africa.

The basic reproduction number in our model depends on the initial exposure rates including exposure to cadavers of EVD victims. The provision of scope for further quarantining during the progression of the infection means that these exposure rates are weighted accordingly, depending on whether or not the system woke up from slumber and picked up those persons who initially escaped quarantine. For example, the parameter *τ*_*Q*_ which measures the effective contact rate between confirmed quarantined early symptomatic individuals and susceptible individuals is eventually scaled by the proportions *θ*_3*a*_ and *θ*_2*a*_ which, respectively, are those proportions of suspected and probable cases that eventually progress to become EVD patients and have escaped quarantine. Thus our framework can progressively be used at each stage to manage the progression of infections in the community.

Our results show that eventually the system settles down to a nonzero fixed point when there is constant recruitment into the population of 555 persons per day and for *R*_0_ > 1. The values of the steady states are completely determined in terms of the parameters in this case. Our analysis also shows that it is possible to control EVD infection in the community provided we reduce and maintain the reproduction number to below unity. Such control measures are possible if there is effective contact tracing and identification of EVD patients and effective quarantining, since a reduction of the proportion of cases that escape quarantine reduces the value of *R*_0_.

Additionally, our model results indicate that when there are a high constant number of recruitment into an EVD community, quarantining alone may not be sufficient to eradicate the disease. It may serve as a buffer enhancing a sustained epidemic. However, reducing the number of persons recruited per day can bring the diseases to very low values.

To demonstrate the feasibility of our results, we performed a pseudoequilibrium approximation to the system derived based on the assumption that the duration of manifestation of EVD infection in the community, per individual, is short when compared with the natural life span of an average human. The reduced model was used to show that all steady state solutions are stable to small perturbations and that there can be oscillatory returns to the equilibrium solution. These results were confirmed with numerical simulations. Given the size of the system, we have not been able to perform a detailed nonlinear analysis on the model. However the discussion on the nature of the parameters for the model is based on the statistics gathered from the 2014 EVD outbreak in Africa and we believe that our model can be useful in characterizing and studying a class of epidemics of Ebola-type. We have not yet carried out a complete sensitivity analysis on all the parameters to determine the most crucial parameters in our model. This is under consideration. Furthermore, the effect of stochasticity seems relevant to study. This and other aspects of the model are under consideration.

## Figures and Tables

**Table 1:** A possible progression path of symptoms from  exposure to the Ebola Virus to treatment or death. Table shows a suggested transition and time frame in humans, of the virus, from exposure to incubation to symptoms development and recovery or death. This table is adapted based on the image in the Huffington post, via [11]. Superscript a: for the 2014 epidemic, the average incubation period is reported to be between 9 and 11 days [10]. Superscript b: other studies reported a mean of 4–10 days [8, 9].

Exposure	Incubation period	Course of illness			Recovery or death	
	Range: 2 to 21 days from exposure	Range: 6 to 16 days from the end of the incubation period			Recovery: by the end of days 6–11	Death: by the end of days 6–16
		Probable	Early symptomatic	Late symptomatic		
		Days 1–3	Days 4–7	Days 7–10		
An individual comes in contact with an Ebola infected individual (dead or alive) or have been in the vicinity of someone who has been exposed.	Average of 8–11^a^ days before symptoms are evident. Another estimate reports an average of 4–10^b^ days.	Patients exhibit malaria-like or flu-like symptoms: for example, fever and weakness.	Patients progress to gastrointestinal symptoms: for example, nausea, watery diarrhea, vomiting, and abdominal pain. Other symptoms may include low blood pressure, anemia, headaches, chest pain, shortness of breath, exhibition of a rash, confusion, bleeding, and conjunctivitis.	Patients may present with confusion and may exhibit signs of internal and/or visible bleeding, potentially progressing towards coma, shock, and death.	Some patients may recover, while others will die. Recovery typically requires early intervention.	

**Table 2:** Parameters, baseline values, and ranges of baseline values with references.

Parameters	Baseline values	Range of values	Reference
Π	3, 555	Varies	
*ρ*_*N*_	Varies	Varies	
*ρ*_*Q*_	Varies	Varies	
*ξ*_*N*_	0.27	[0.12,0.48]	Estimated^*∗*^
*τ*_*N*_	*q*_*N*_ *ξ*_*N*_, *q*_*N*_ = 0.75	*q*_*N*_ ∈ [0,1]	Variable
*ξ*_*Q*_	*ϕ*_*Q*_ *ξ*_*N*_, *ϕ*_*Q*_ = 0.5	*ϕ*_*Q*_ ∈ [0,1]	Estimated^*∗*^
*τ*_*Q*_	*q*_*Q*_ *ξ*_*Q*_, *q*_*Q*_ = 0.75	*q*_*Q*_ ∈ [0,1]	Variable
*θ*_*i*_	*θ*_*i*_ ∈ [0,1], *i* = 1,2,…, 7	*θ*_*i*_ ∈ [0,1], *i* = 1,2,…, 7	Variable
*α*_*N*_	1/10	[1/11,1/4]	[8–10]
*α*_*Q*_	1/10	[1/11,1/4]	[8–10]
*β*_*N*_	0.5	[1/3,1]	[10]
*β*_*Q*_	1/2	[1/3,1]	[10]
*γ*_*N*_	1/3	[1/4,1/2]	[10, 12]
*γ*_*Q*_	1/3	[1/4,1/2]	[10, 12]
*δ*_*N*_	1/3	[1/4,1/2]	[10, 12]
*δ*_*Q*_	1/3	[1/4,1/2]	[10, 12]
*μ*	1/(60 × 365)	[1/(40 × 365), 1/(70 × 365)] day^−1^	[13]
*b*	1/2.5	[1/4.50,1/2] day^−1^	[14, 15]
*a*_*D*_	0.3000	[0.111,0.489] day^−1^	[15]
*r*_*EQ*_	0.5366	[0.4829,0.5903]	[16]
*r*_*EN*_	*ωr*_*EQ*_, *ω* = 0.88	*ω* ∈ [0,1]	Estimate
*r*_*LN*_	*κr*_*EN*_, *κ* = 0.02	*κ* ≪ 1	Estimate
*r*_*LQ*_	*κr*_*EQ*_, *κ* = 0.02	*κ* ≪ 1	Estimate
*σ*_*N*_	0.5	[1/3,1]	Estimate

^*∗*^Estimates discussed in Section 4.

## Notations

### State Variables and Their Descriptions

*H*_*L*_(*t*): Total population density of living humans at any time *t*
*H*(*t*): The total population density at time *t* of living humans together with Ebola-related cadavers, that is, those humans who have died of EVD and have not yet been disposed at time *t*
*S*(*t*): Population density at time *t* of all susceptible humans in the population
*S*_*N*_(*t*), *S*_*Q*_(*t*): Population densities at time *t* of all humans that are known to have been in contact with or have a history of association with any person known to have once had or died of EVD; these are suspected Ebola Virus patient cases that are either not quarantined *S*_*N*_ or quarantined *S*_*Q*_ and who are not yet showing any Ebola-like symptoms
*P*_*N*_(*t*), *P*_*Q*_(*t*): Population densities at time *t* of all persons suspected of having EVD infection and who present with fever and at least three other Ebola-like symptoms; these probable cases are either not quarantined *P*_*N*_ or quarantined *P*_*Q*_
*C*_*N*_(*t*), *C*_*Q*_(*t*): Population densities at time *t* of all probable Ebola Virus infected humans who after a lab test have been confirmed to indeed have EVD infection and who still present only early Ebola-like symptoms of fever, aches, tiredness, and so forth; they are called confirmed early symptomatic in the sense explained in the text; these confirmed Ebola Virus carriers are either not quarantined *C*_*N*_ or quarantined *C*_*Q*_
*I*_*N*_(*t*), *I*_*Q*_(*t*): Population densities at time *t* of all confirmed EVD patients who now present with full later stage Ebola-like symptoms; they are called confirmed late symptomatic in the sense explained in the text; these confirmed EVD patients with full blown symptoms are either not quarantined *I*_*N*_ or quarantined *I*_*Q*_
*R*_*R*_(*t*): Population density at time *t* of all humans who were once infected with EVD infection and who have recovered from the infection; this class of persons are then immune to any further infection and are removed from the susceptible pool
*R*_*D*_(*t*): Population density at time *t* of all humans who were once infected with EVD and who have died because of the EVD infection; this class of persons though dead are still infectious
*R*_*N*_(*t*): Population density at time *t* of all humans who died naturally or due to other causes; this is just a collection class
*D*_*D*_(*t*): Population density at time *t* of all Ebola-related dead humans removed from the infection cycle because they received proper burial or were cremated.

### Parameters, Their Descriptions, and Their Corresponding Quasidimension

Π: Net constant migration rate of humans, *H*_*L*_ *T* ^−1^
*ρ*_*N*_: Effective contact rate between probable nonquarantined individuals and susceptible individuals; a fraction *θ*_3_ of these contacts are potentially infectious to the susceptible humans, *T* ^−1^
*ρ*_*Q*_: Effective contact rate between probable quarantined individuals and susceptible individuals; a fraction *θ*_7_ of these contacts are potentially infectious to the susceptible humans, *T* ^−1^
*τ*_*N*_: Effective contact rate between confirmed nonquarantined early symptomatic individuals and susceptible individuals, *T* ^−1^
*ξ*_*N*_: Effective contact rate between confirmed nonquarantined late symptomatic individuals and susceptible individuals, *T* ^−1^
*τ*_*Q*_: Effective contact rate between confirmed quarantined early symptomatic individuals and susceptible individuals, *T* ^−1^
*ξ*_*Q*_: Effective contact rate between confirmed quarantined late symptomatic individuals and susceptible individuals, *T* ^−1^
*a*_*D*_: Effective contact rate between cadavers of confirmed late symptomatic individuals and susceptible individuals, *T* ^−1^
*λ*: A real function depending on the active members of the population representing the force of infection, *T* ^−1^
*θ*_*i*_: Proportions; 0 ≤ *θ*_*i*_ ≤ 1, *i* = 1,2,…, 7, 1
*α*_*N*_: Rate at which nonquarantined suspected cases become nonquarantined probable cases, *T* ^−1^
*α*_*Q*_: Rate at which quarantined suspected cases become quarantined probable cases, *T* ^−1^
*β*_*N*_: Rate at which nonquarantined probable cases become nonquarantined confirmed early symptomatic cases, *T* ^−1^
*β*_*Q*_: Rate at which quarantined probable cases become quarantined confirmed early symptomatic cases, *T* ^−1^
*γ*_*N*_: Rate at which nonquarantined confirmed early symptomatic cases become nonquarantined confirmed late symptomatic cases, *T* ^−1^
*γ*_*Q*_: Rate at which quarantined confirmed early symptomatic cases become quarantined confirmed late symptomatic cases, *T* ^−1^
*δ*_*N*_: Rate at which nonquarantined confirmed late symptomatic cases die due to the EVD, *T* ^−1^
*δ*_*Q*_: Rate at which quarantined confirmed late symptomatic cases die due to the EVD, *T* ^−1^
*μ*: Constant natural death rate for humans, *T* ^−1^
*b*: Rate at which cadavers are removed and buried, *T* ^−1^
*r*_*EN*_: Rate of recovery of nonquarantined confirmed early symptomatic cases, *T* ^−1^
*r*_*LN*_: Rate of recovery of nonquarantined confirmed late symptomatic cases, *T* ^−1^
*r*_*EQ*_: Rate at which quarantined confirmed early symptomatic cases recover, *T* ^−1^
*r*_*LQ*_: Rate at which quarantined confirmed late symptomatic cases recover, *T* ^−1^
*σ*_*N*_: Rate at which nonquarantined confirmed late symptomatic cases are removed and quarantined, *T* ^−1^.
